# Injury Activates a Dynamic Cytoprotective Network to Confer Stress Resilience and Drive Repair

**DOI:** 10.1016/j.cub.2019.09.035

**Published:** 2019-11-18

**Authors:** Helen Weavers, Will Wood, Paul Martin

**Affiliations:** 1 School of Biochemistry, Biomedical Sciences, University of Bristol, Bristol BS8 1TD, UK; 2 School of Cellular and Molecular Medicine, Biomedical Sciences, University of Bristol, Bristol BS8 1TD, UK; 3 Centre for Inflammation Research, University of Edinburgh, Queens Medical Research Institute, Edinburgh EH16 4TJ, UK; 4 School of Physiology, Pharmacology and Neuroscience, Biomedical Sciences, University of Bristol, Bristol BS8 1TD, UK; 5 School of Medicine, Cardiff University, Cardiff CF14 4XN, UK

**Keywords:** inflammation, tissue repair, tissue resilience, cytoprotection, oxidative stress, ROS, DNA damage, hormesis, wound, preconditioning

## Abstract

In healthy individuals, injured tissues rapidly repair themselves following damage. Within a healing skin wound, recruited inflammatory cells release a multitude of bacteriocidal factors, including reactive oxygen species (ROS), to eliminate invading pathogens. Paradoxically, while these highly reactive ROS confer resistance to infection, they are also toxic to host tissues and may ultimately delay repair. Repairing tissues have therefore evolved powerful cytoprotective “resilience” machinery to protect against and tolerate this collateral damage. Here, we use *in vivo* time-lapse imaging and genetic manipulation in *Drosophila* to dissect the molecular and cellular mechanisms that drive tissue resilience to wound-induced stress. We identify a dynamic, cross-regulatory network of stress-activated cytoprotective pathways, linking calcium, JNK, Nrf2, and Gadd45, that act to both “shield” tissues from oxidative damage and promote efficient damage repair. Ectopic activation of these pathways confers stress protection to naive tissue, while their inhibition leads to marked delays in wound closure. Strikingly, the induction of cytoprotection is tightly linked to the pathways that initiate the inflammatory response, suggesting evolution of a fail-safe mechanism for tissue protection each time inflammation is triggered. A better understanding of these resilience mechanisms—their identities and precise spatiotemporal regulation—is of major clinical importance for development of therapeutic interventions for all pathologies linked to oxidative stress, including debilitating chronic non-healing wounds.

## Highlights

• Tissue damage and inflammation activate cytoprotective genes to confer resilience
• Nrf2 and Gadd45 resilience factors are required for effective wound repair *in vivo*
• Nrf2 and Gaddd45 limit inflammatory ROS damage and promote DNA damage repair
• Ectopic resilience gene induction can protect naive unwounded tissue from damage

## Graphical Abstract

**Figure:**
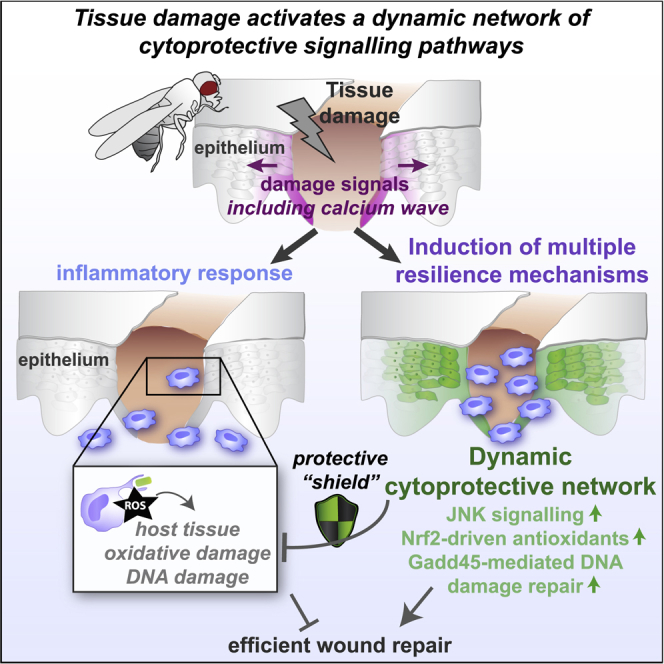

## Teaser

Weavers et al. use live-imaging and genetic approaches in *Drosophila* to identify how tissue damage activates a dynamic cytoprotective network (involving JNK, Nrf2, and Gadd45) within the repairing epithelium, which confers stress “resilience” by protecting against inflammatory ROS damage, and is essential for driving efficient wound repair.

## Introduction

Reactive oxygen species (ROS) are universal injury-induced signals, produced by NADPH oxidases as an immediate response to tissue damage [1]. At low levels, ROS can function as attractants for the recruitment of innate immune cells [2, 3] and to promote efficient wound angiogenesis [4]; however, incoming inflammatory cells generate additional ROS in a “respiratory burst” to eliminate invading pathogens and confer resistance to infection [5, 6]. Although this bacteriocidal response is clearly beneficial, excessive ROS levels can cause substantial bystander damage to host tissue [5]; indeed, excessive oxidative stress is thought to be a key player in the pathogenesis of chronic non-healing wounds of patients in the clinic [7, 8, 9].

To counter inflammatory stress, host tissues must employ powerful cytoprotective machinery to limit the “collateral” damage and prevent immunopathology [10]. Mammalian wound studies have identified a number of signaling pathways that may promote protection against oxidative stress [11, 12], but such investigations have been complicated by the intricacy of the protection machinery and relative genetic intractability of vertebrate models. Nevertheless, a better understanding of these protective mechanisms will be crucial to enable the development of improved therapeutic interventions for a wide range of oxidative stress-related diseases, including chronic non-healing wounds. Also in the context of wound repair, therapeutic activation of cytoprotective pathways in the clinic could also offer an exciting approach to “precondition” patient tissues prior to elective surgery [13].

Here, we develop a novel experimentally amenable *Drosophila* model in which to dissect the complex cytoprotective mechanisms that render repairing tissues “resilient” to inflammation-derived damage. *Drosophila* is a well-established model for uncovering fundamental, conserved aspects of wound repair and the inflammatory response [14, 15, 16] and offers unrivalled genetic tractability and optical translucency for high-resolution *in vivo* imaging.

In this study, we characterize the temporal and spatial dynamics of the stress “resilience” mechanisms that are induced downstream of wounding and dissect the underlying molecular and cellular mechanisms driving tissue protection. We identify a complex cross-regulatory network of cytoprotective pathways, involving calcium, JNK, Nrf2, and Gadd45, which collectively “shield” tissues from ROS-induced damage and promote efficient damage repair. RNAi-mediated inhibition of either Nrf2 or Gadd45 delays wound repair, which is further exacerbated if both pathways are inhibited. Interestingly, we find that these cytoprotective pathways are activated downstream of the same calcium signaling pathway that initiates the inflammatory response, suggesting the existence of a “fail-safe” mechanism for cytoprotection whenever inflammation is triggered. Finally, ectopic activation of these protective pathways can confer stress resilience to naive unwounded tissue, and in the case of Gadd45, can even accelerate the rate of wound repair. Prolonged activation of Nrf2, however, caused marked delays in wound repair, suggesting that the optimal level of cytoprotection required for the most efficient tissue repair will be a finely tuned spatiotemporal balance of cytoprotective signaling.

## Results

### Tissue Damage Triggers a Burst of Inflammatory ROS and ROS-Induced Damage

**Figure 1:**
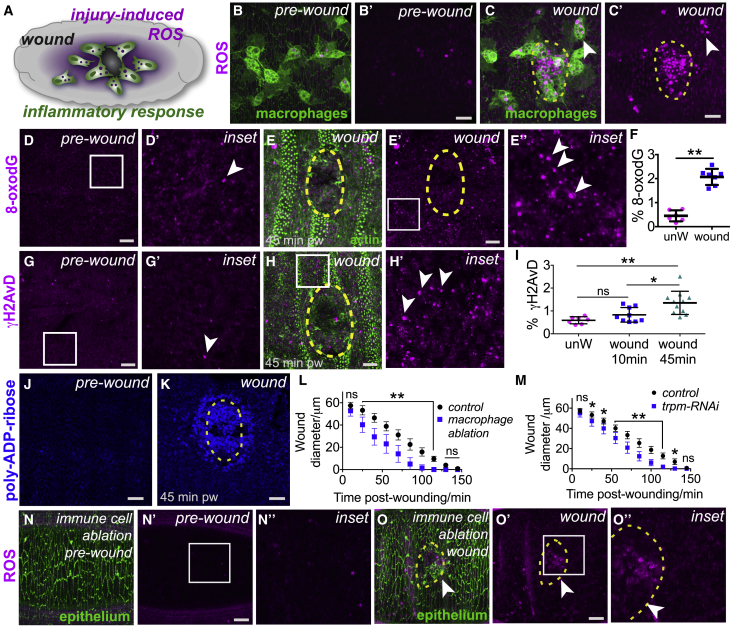
Wound-Induced Inflammation Triggers ROS Production and Oxidative Damage (A–K) Wounding and inflammation (green immune cells, *srp > GFP*, A–C) in *Drosophila* embryos are associated with increased ROS (magenta, DHE staining) production (A, schematic; B, pre-wound; C, 1 h post-wound; arrowheads indicate ROS within immune cells), oxidative damage (arrowheads, magenta 8-oxo-dG, D and E; quantified in F), γH2AvD puncta (arrowheads, magenta, G and H; quantified in I), and PARylation (blue, J and K). % 8-oxo-dG and % γH2AvD refer to percent (%) of area measured that is positive for marker of interest after thresholding. (L–O) Inhibition of wound inflammation (macrophage ablation using *srp > reaper* [L] and *trpm-RNAi* [M]) accelerates the rate of wound closure compared to controls (quantified in L and M, n > 20 for each condition). Macrophage ablation is associated with reduced ROS production (magenta DHE staining) before (N) and after (arrowheads, O) wounding compared to controls (B and C). Wound edge represented by dashed yellow outlines in (C), (E), (H), (K), and (O). Scale bars represent 10 μm in (B)–(E), (G), (H), (J), (K), (N), and (O). Data represented as mean ± SEM; ^∗^p < 0.05 and ^∗∗^p < 0.01 via the Mann-Whitney Test (F), one-way ANOVA followed by Dunn’s multiple comparisons test (I), or multiple t tests followed by Holm-Sidak multiple comparisons correction (L and M). See also Figure S1 and Video S1.

A dramatic increase in ROS levels occurs during the inflammatory wound response (Video S1) within *Drosophila* embryos (Figures 1A–1C; higher magnification views, Figures S1A–S1G) [2]; this is accompanied by a significant increase in levels of oxidative DNA damage (base adduct 8-oxo-dG; Figures 1D and 1E; quantified in Figure 1F) and activation of the DNA damage response within epithelial cells at the wound margin (γH2AvD, the *Drosophila* ortholog of mammalian γH2AX; Figures 1G and 1H; quantified in Figure 1I; PARylation, Figures 1J and 1K). These DNA damage markers (PAR, 8-oxo-dG, and γH2AvD) are highly responsive to ROS levels, as shown by exposure to exogenous H_2_O_2_ or ectopic expression of the antioxidant enzyme Catalase (Figures S1H–S1V). The high levels of ROS and oxidative damage suggest that the wound-induced inflammatory response (despite being necessary to fight potential infection) might also be detrimental to tissue repair. We therefore inhibited wound inflammation, either by genetic ablation of immune cells (termed “hemocytes” in *Drosophila*, using *srp-Gal4*-driven expression of the pro-apoptotic gene *reaper*; Figures 1L, S1W, and S1X) or by blocking propagation of the pro-inflammatory calcium wave [17] (using RNAi-mediated inhibition of the Trpm calcium channel; Figures 1M, S1Y, and S1Z); in both cases, wound closure was accelerated in the absence of inflammation (Figures 1L and 1M). Detailed analysis of wound closure indicates that, while the rapidly assembled actin cables at the wound leading edge appeared indistinguishable from controls (insets, Figures S1Y and S1Z), the repairing epithelial sheet migrated faster than normal to seal the wound—suggesting that inflammatory ROS may normally impede cell migration. Indeed, ROS production was significantly reduced following immune cell ablation (Figures 1N and 1O) compared to controls.

Video S1. *In Vivo* Time-Lapse Imaging of Wound Repair and Inflammation within *Drosophila* Embryos, Related to Figure 1 *in vivo* time-lapse video within a wounded *Drosophila* embryo, with time-points every 30 s. Within minutes of injury, epithelial cells (magenta, labeled with *moesin-GFP*) within the leading edge of the wound assemble an actin cable and, together with leading edge actin-rich protrusions, this helps close the wound. In parallel to wound repair, *Drosophila* hemocytes (green, labeled by *srp-Gal4* driven *moesin-mcherry*) are rapidly recruited to the site of injury and accumulate in high numbers at the wound site. Scale bar represents 10 μm.

### Wounding Induces a Zone of Stress Resilience within the Repairing Epithelium

**Figure 2:**
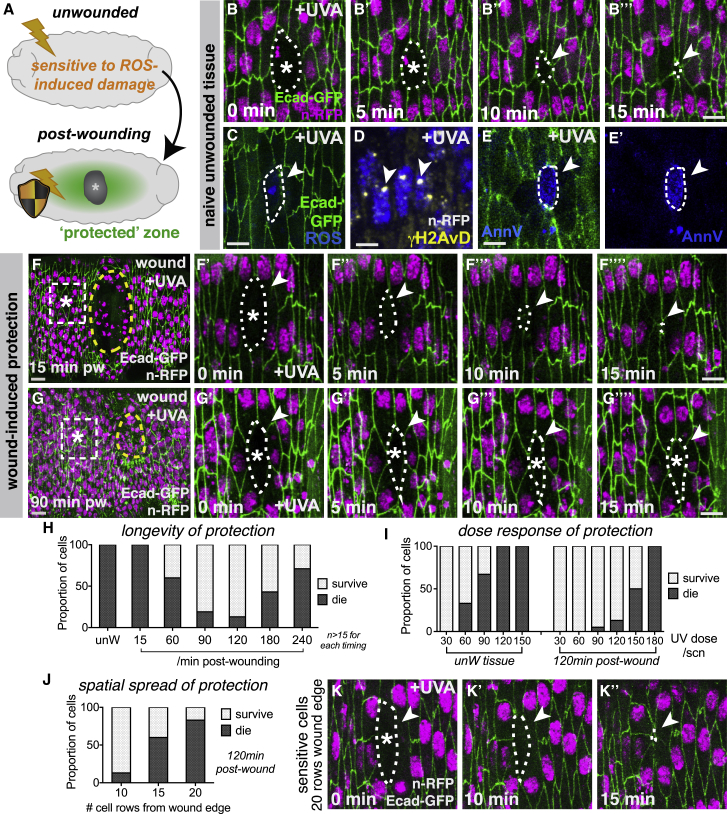
Tissue Damage Activates a Transient Zone of Stress Resilience within the Repairing Epithelium (A–E) Wounding triggers the epithelium to become more resistant to UV-induced cell damage and death (schematic, A). Naive unwounded tissue is sensitive to UV-A (B–E), with targeted cells (asterisks) rapidly rounding up and delaminating (B; magenta nuclei, *His2Av-mRFP*; green cell outlines, *dE-cadherin-GFP*), with increased ROS (blue DHE in C), γH2AvD puncta (arrowheads, yellow in D), and AnnexinV staining (arrowheads, blue, E and E′). (F and G) Wounded epithelium (magenta nuclei, *His2Av-mRFP*; green cell outlines, *dE-cadherin-GFP*) initially sensitive to UV-A (F) with targeted cells (asterisks) delaminating from epithelium (arrowheads, F′–F″″) as in controls. Wounded epithelium more resistant to UV-induced stress 90 min post-wounding (G) with targeted cells (asterisk) remaining in epithelium (arrowheads, G′–G″″). (H–K) The induction of UV resistance is temporary (quantified in H), displays a typical dose-response behavior (quantified in I), and fades with increasing distance from the wound edge (J and K). Wound edge represented by dashed yellow outlines in (F) and (G); UV-targeted cells indicated by dashed white line in (B), (C), (E), (F′)–(F″″), (G′)–(G″″), and (K)–(K″). pw, post-wounding. Scale bars represent 10 μm in (F) and (G) and 5 μm in (B)–(E), (F′)–(F″″), (G′)–(G″″), and (K)–(K″). See also Figure S2 and Videos S2 and S3.

Given the marked increase in ROS production and oxidative damage following wounding, it is perhaps somewhat surprising that only minimal levels of apoptosis are normally observed around healthy wounds with a standard robust inflammatory response (our previous work) [15]. To explain this, we envision that injured tissues might normally activate protective pathways to counter inflammation-associated damage. To investigate such a phenomenon, we developed a proxy model to test the sensitivity of the wounded epithelium to stress (Figure 2), using micro-irradiation with UV-A light. Individual cells within the “naive” unwounded epithelium of *Drosophila* embryos are highly sensitive to UV-A-induced damage and rapidly undergo apoptosis (Figures 2A–2E; Video S2) [15]. UV-A induced ROS production within the targeted cells (Figures 2C and S2A), and this is associated with an increase in a variety of DNA lesions, including the oxidative base adduct 8-oxo-dG (Figure S2B), poly-ADP-ribose (Figure S2C), and double-strand DNA breaks (γH2AvD; compare Figure 2D with Figure 1G′). Individual UV-damaged cells rapidly delaminate from the epithelium (Figure 2B) while exhibiting positive AnnexinV staining on their surface (Figures 2E and S2D) and are rapidly engulfed by migrating macrophages (Figure S2E) [15]. Such apoptotic stress responses are generally considered critical fail-safe mechanisms to prevent malignant transformation, with excessive unrepaired DNA damage and high levels of ROS leading to activation of death-receptor signaling [18].

Video S2. Cells Within the “Naive” Unwounded Epithelium of *Drosophila* Embryos Are Highly Sensitive to UVA-Induced Damage, Related to Figure 2 *in vivo* time-lapse videos of UVA-induced epithelial cell death and delamination (left) which is accompanied by increased ROS production (center) and AnnexinV staining (right) within the targeted cells. A brief pulse of UVA targeted on individual or a small group of epithelial cells causes the targeted cells to round up and delaminate from the epithelium (left; magenta nuclei labeled with nuclear RFP and green cell outlines labeled with GFP-tagged E-cadherin, with time-points every 1min). Following UVA exposure, targeted cells experience an increase in ROS levels (center; ROS, blue, stained with DHE and green cell outlines labeled with GFP-tagged E-cadherin, with time-points every 1min) and exhibit elevated AnnexinV (AnnV) staining on their surface (right; AnnV, blue, and green epithelial cells labeled with GFP-Moesin, with time-points every 1min). Scale bar represents 5 μm.

Strikingly, we find that the epithelium of wounded embryos develops increased resistance to UV-induced apoptosis in a strict spatiotemporal manner following injury (Figure 2A). Individual epithelial cells in the vicinity of the wound, if targeted with UV-A within the first 30 min post-wounding, display similar sensitivity to those within an unwounded epithelium, rapidly rounding up and delaminating basally (Figure 2F; Video S3), with removal by macrophages (Figure S2F). However, with increasing time post-wounding, these cells display a striking change in UV-A sensitivity (Figures 2G–2J). From 60 min post-wounding onward, cells extending back up to 10 cell diameters from the wound margin within the repairing epithelium become more resistant to the UV-A-induced apoptosis and often fail to delaminate (Figure 2G; quantified in Figure 2H and Video S3). The proportion of cells exhibiting this UV resistance increases until 120 min post-wounding, but the protective effect is temporary, and UV resistance steadily declines from 3 h post-wounding onward (Figure 2H). The response of targeted cells to UV exposure followed a typical “dose-response” relationship, with increased UV exposure times inducing a progressively higher proportion of epithelial cell death for both unwounded and wounded tissues (Figure 2I); nevertheless, cells around the wound edge could resist significantly higher UV doses than cells of unwounded controls (Figure 2I).

Video S3. Wounding Confers Increased Resistance to Stress-Induced Cell Death, Related to Figure 2 *in vivo* time-lapse videos illustrating the increased resistance to stress-induced cell death that is observed within the repairing epithelium following wounding. Epithelial cells within the repairing epithelium initially remain sensitive to UVA-induced cell death (asterisk), in a similar manner to that observed within the unwounded epithelium (left, 15min post-wounding; magenta nuclei labeled with nuclear RFP and green cell outlines labeled with GFP-tagged E-cadherin). With increased time post-wounding, cells within the repairing epithelium become less sensitive to UVA-induced death and UVA-targeted cells (asterisk) remain within the epithelial layer (right, 90min post-wounding). In all videos, time-points are shown every 1min. Scale bars represent 5 μm.

The protective effect spreads outward from the wound margin and declines with increasing distance from the wound edge (quantified in Figure 2J) with only minimal protection observed at 20 cells from the wound edge (at 120 min post-wounding) with the majority of cells delaminating after UV exposure (Figure 2K). Epithelial cells targeted with UV within the protected zone that fail to delaminate are also ignored by nearby macrophages (Figure S2G), suggesting the targeted epithelial cells fail to display normal apoptotic “eat me” signals. Intriguingly, cells targeted at an intermediate time point (approximately 45 min post-wounding) display a surprising transitional behavior, initially rounding up (as in unwounded tissues) but then recovering and remaining within the epithelium (Figure S2H), with no associated recognition by nearby macrophages (Figure S2I); it is possible these epithelial cells are able to recover from the “brink of death” similar to that observed recently within certain tissues during *Drosophila* development [19]. These data suggest that cells in the vicinity of repairing epithelial tissues dynamically upregulate protective mechanisms following wounding, which make them more *resilient* to stress-induced cell damage or death.

### Wounding Activates Multiple Cytoprotective Pathways

**Figure 3:**
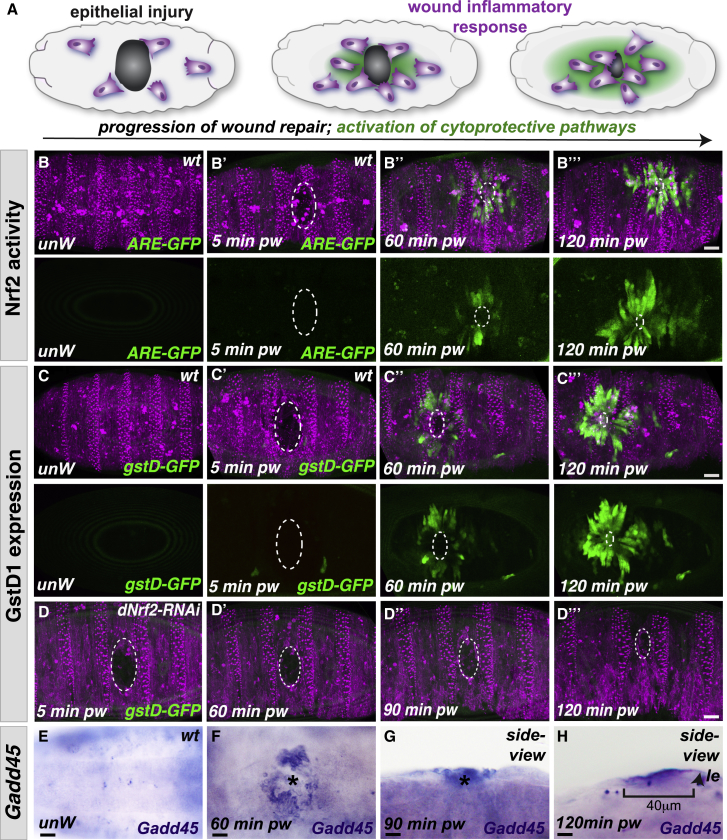
Multiple Cytoprotective Pathways Activated Downstream of Wounding (A) Epithelial wounding and inflammation (magenta) in *Drosophila* embryos trigger the activation of multiple cytoprotective pathways (green, schematic). (B) Nrf2 signaling (green, ARE-GFP reporter), which is absent from the epithelium (magenta, ubiquitous Moesin-mCherry) of control unwounded embryos, is activated in a wave spreading out from the wound site. (C) GstD1 expression (green, gstD-ARE:GFP transgenic reporter) is also absent from control unwounded embryos (magenta epithelium, ubiquitous Moesin-mCherry), but GstD1 expression increases in a similar wave pattern spreading out from the wound. (D) Wound-induced activation of GstD1 expression (green, *GstD-GFP* reporter) within the repairing epithelium is lost following RNAi-mediated inhibition of *dNrf2* expression compared to controls (C). (E–H) *Gadd45* expression (purple, *in situ* hybridization, E–H) is also undetectable in control unwounded epithelium (E) but increases in the repairing epithelium following wounding (F, ventral view; G and H, lateral views). *Gadd45* expression extends up to 40 μm back from the wound leading edge (le, arrowhead) within the repairing epithelium 120 min following wounding (H). pw, post-wounding; le, leading edge. Scale bars represent 15 μm in (B)–(D) and 10 μm in (E)–(G). See also Videos S4 and S5.

We next investigated which stress-induced pathways could be responsible for driving wound-induced “resilience.” The temporal dynamics of protection induction suggest that resilience is likely to, at least in part, require *de novo* transcription or translation. In fact, we find that multiple genes with potential cytoprotective activity are upregulated within the wounded epithelium, with strikingly similar spatiotemporal dynamics to the induction of UV-A resilience (Figure 3A). Nrf2 is a master regulator of the cellular antioxidant response [20] and is transcriptionally activated within mammalian wounds [21]. Using an *in vivo* fluorescence reporter of *Drosophila* Nrf2 activity (Cap “n” collar isoform-C, CncC [22]) [23], we live-imaged the spatiotemporal dynamics of Nrf2 signaling upon wounding (Figure 3B; Video S4) and observed a wave of Nrf2 activity spreading out from the wound margins. As in mammals, *Drosophila* Nrf2 activity can also be regulated at the post-translational level by its binding partner and inhibitor Keap1 [24]. Oxidative stress is known to inactivate Keap1, allowing subsequent Nrf2 stabilization and activation of Nrf2 signaling [25]; a similar oxidative stress-mediated inhibition of Keap1 post-wounding could activate Nrf2 post-translationally in our system. We observe a similar, wound-induced, wave-like expression pattern upon wounding for *Drosophila* GstD1 (Figure 3C; Video S5), a glutathione S transferase (GST) enzyme involved in glutathione-mediated detoxification and a known target of Nrf2 [22]. Consistent with this, we find that *dNrf2-RNAi* expression abolishes the wound-triggered upregulation of *GstD-GFP* (Figure 3D), suggesting that Nrf2 and its downstream targets may confer tissue resilience post-wounding (perhaps via ROS detoxification).

Video S4. Injury Triggers dNrf2 Activation within the Repairing Epithelium, Related to Figure 3 *in vivo* time-lapse videos of *Drosophila* Nrf2 activation within the repairing epithelium of *Drosophila* embryos. Nrf2 activity (green, *ARE-GFP* reporter) is absent from unwounded embryos but increases within the repairing epithelium (upper panel; magenta, labeled within Moesin-mCherry) following wounding (asterisks), spreading out from the wound edge in a wave-like manner, with time-points every 7min. Scale bars represent 10 μm.

Video S5. Injury Triggers GstD1 Expression within the Repairing Epithelium, Related to Figure 3 *in vivo* time-lapse imaging of GstD1 levels within the repairing epithelium of *Drosophila* embryos. GstD1 levels (green, *gstD1-GFP* reporter) are undetectable within the unwounded epithelium but increase dramatically within the repairing epithelium (magenta, labeled within Moesin-mCherry) following wounding, in a similar manner to that observed for Nrf2 activity, spreading out from the wound edge in a wave-like manner, with time-points every 7min. Scale bars represent 10 μm.

However, we envision that wounded tissues will upregulate a multitude of further protection strategies that target different cellular components and act collectively to reduce damage. Indeed, we find that *Drosophila* Gadd45 (the single fly homolog of the mammalian growth arrest and DNA-damage inducible GADD45 gene family [26]) is transcriptionally induced within the *Drosophila* wounded epithelium with strikingly similar spatiotemporal dynamics (Figures 3E–3H) [27] to Nrf2 activity. Since Gadd45 has been implicated in DNA damage repair in both mammals and flies [28, 29], it could mediate an additional level of protection by promoting repair of DNA damage induced by ROS that escaped Nrf2-mediated detoxification.

### Nrf2 and Gadd45 Confer Resilience to Naive Tissues and Are Required for Wound Repair

**Figure 4:**
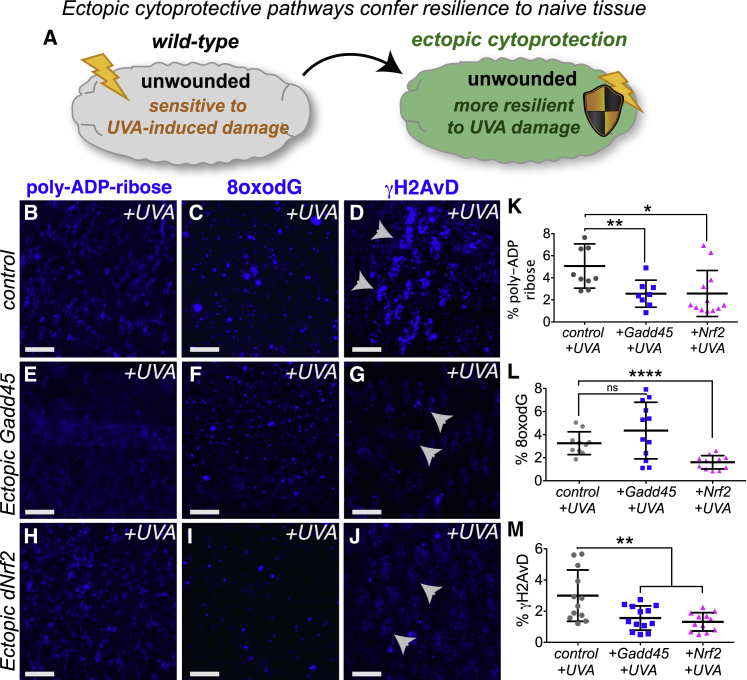
Wound-Induced Pathways Can Confer Protection against Oxidative Damage to Naive Unwounded Tissue Ectopic expression of either *Gadd45* (A, schematic and E–G) or *dNrf2* (H–J) in unwounded *Drosophila* embryos confers increased protection against UV-induced damage compared to controls (B–D), as shown by poly-ADP-ribose, 8-oxo-dG, and γH2AvD staining (quantified in K–M). Arrowheads (D, G, and J) indicate punctae of γH2AvD staining. % poly-ADP-ribose (PAR), % 8-oxo-dG, and % γH2AvD refer to percent (%) of area measured that is positive for marker of interest after thresholding. Scale bars represent 5 μm in (B)–(J). Data represented as mean ± SEM; ns, not significant, ^∗^p < 0.05 and ^∗∗^p < 0.01 via multiple t tests followed by Holm-Sidak multiple comparisons correction (K–M).

To determine whether Nrf2 and Gadd45 promote tissue resilience to stress, we tested whether their ectopic activation could confer protection to naive unwounded tissues (Figures 4A–4M). Using the GAL4-UAS system [30] for genetic mis-expression, we find that ectopic expression of *Gadd45* (Figures 4E–4G) or *dNrf2* (Figures 4H–4J) indeed confers stress resilience to cells within the unwounded epithelium of *Drosophila* embryos compared to controls (Figures 4B–4D). Unlike the high levels of DNA damage (as detected by PARylation [Figure 4B] and γH2AvD [Figure 4D]) induced by UV-A irradiation of control cells, cells with elevated Gadd45 expression exhibit remarkable resistance to UV-A-induced DNA damage (Figures 4E and 4G; quantified in Figures 4K and 4M, respectively), although levels of oxidative damage (as detected by 8-oxo-dG) remain indistinguishable from controls (Figures 4C, 4F, and 4L). Epithelial cells with elevated Nrf2 levels also exhibit significantly less damage than control UV-A-irradiated cells (as detected by PARylation, 8-oxo-dG, and γH2AvD; Figures 4H–4J; quantified in Figures 4K–4M). Interestingly, however, ectopic Nrf2 alone wasn’t sufficient to completely protect these cells from UV-A-induced death (data not shown), suggesting that full stress protection (as observed upon wounding) requires the activity of multiple cytoprotective pathways.

**Figure 5:**
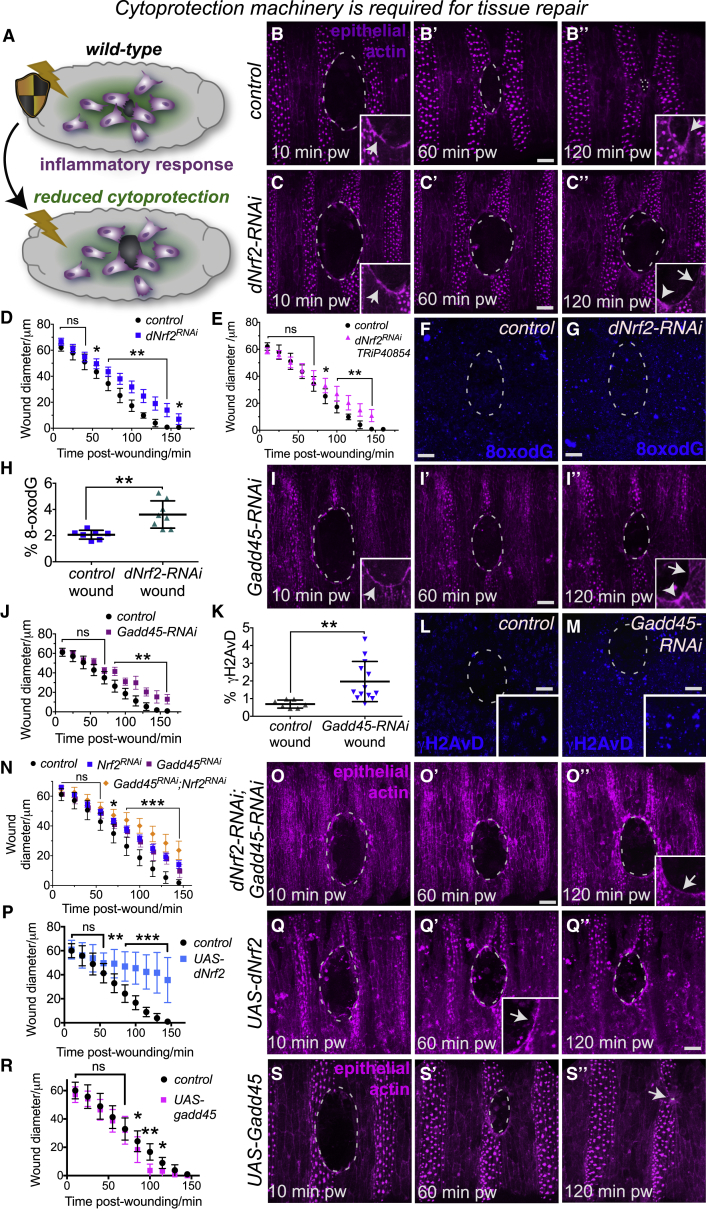
Wound-Induced Cytoprotective Pathways Are Required for Efficient Wound Repair *In Vivo* RNAi-mediated inhibition of either *dNrf2* (A, schematic and B–H; independent *dNrf2-RNAi* lines were used in C and D, as in [22], and E, dNrf2-RNAi TRiP40854) or *Gadd45* expression (I–M) caused a delay in epithelial wound repair (C–C″ and quantified in D and E for *dNrf2-RNAi*; I–I″ and quantified in J for *Gadd45-RNAi*, n > 20 for each condition) compared to controls (B–B″; epithelium labeled using Moesin-mCherry in B, C, and I) despite the initial assembly of actin cables at the wound margin (arrowheads, insets, B, C, and I). By 120 min post-wounding, the actin cable had been lost (insets, C″ and I″) compared to controls (inset, B″). Impaired wound healing was associated with elevated levels of oxidative DNA damage (quantified in H; blue, 8-oxo-dG in F and G) following *dNrf2-RNAi* and elevated γH2AvD punctae (quantified in K; blue, γH2AvD in L and M) following *Gadd45-RNAi*. Simultaneous knockdown of dNrf2 and Gadd45 caused more severe delays in wound repair (N and O; inset in O″ indicates loss of actin cable by 120 min post-wounding). Overexpression of dNrf2 significantly delayed wound repair (P and Q) despite assembly of robust actin cable (inset, Q′), but Gadd45 overexpression slightly accelerated wound closure (R and S). pw, post-wounding. % 8-oxo-dG and % γH2AvD refer to percent (%) of area measured that is positive for marker of interest after thresholding. Scale bars represent 10 μm in all panels. Data represented as mean ± SEM; ns, not significant, ^∗^p < 0.05 and ^∗∗^p < 0.01 via the Mann-Whitney Test (H and K) or multiple t tests followed by Holm-Sidak multiple comparisons correction (D, E, J, N, P, and R). See also Figure S3 and Video S6.

We next tested whether these resilience pathways are required for efficient wound repair *in vivo* (Figures 5A–5O). RNAi-mediated knockdown of *Drosophila* Nrf2 (Figures 5C–5H; using multiple independent RNAi lines) or Gadd45 (Figures 5I–5M) caused significant delays in wound closure compared to controls (Figure 5B; Video S6), despite initial assembly of a robust actin cable at the wound leading edge (insets, Figures 5B, 5C, and 5I). Detailed analysis indicated that the repairing epithelium failed to migrate as fast as controls, and this was accompanied by a breakdown in the actin cable at the leading edge by 120 min post-wounding (insets, Figures 5B″, 5C″, and 5I″). These repair defects were associated with increased levels of DNA damage (Figures 5F–5H and 5K–5M) when compared to that of control wounds, suggesting that Nrf2 and Gadd45 are normally required to protect the repairing epithelium from damage. Interestingly, previous reports suggest that oxidative stress negatively impacts cell migration and cytoskeletal organization in various cell types [31, 32]. qRT-PCR was performed to validate that the *dNrf2-RNAi* and *Gadd45-RNAi* lines effectively knock down their mRNA targets (Figures S3A and S3B). Simultaneous knockdown of both dNrf2 and Gadd45 caused a further delay in wound repair (Figures 5N and 5O), suggesting that Nrf2 and Gadd45 act synergistically to promote tissue repair. Interestingly, loss of Gadd45γ in Medaka fish also rendered embryos far more sensitive to irradiation-induced DNA damage [33], suggesting that Gadd45’s role in stress protection could be conserved in vertebrates.

Video S6. Loss of *Gadd45* or *dNrf2* Delays Wound Repair within *Drosophila* Embryos, Related to Figure 5 *in vivo* time-lapse imaging of wound closure following RNAi-mediated inhibition of *dNrf2* (center) or *Gadd45* (right) compared to controls (left), with time-points every 15min. Unlike control wounds (left; epithelium, magenta, labeled with Moesin-mCherry) that close efficiently within 2 h following injury, RNAi-mediated loss of *dNrf2* (center) or *Gadd45* (right) causes a significant delay in wound closure, with the wounds remaining open 125min post-injury, despite the assembly of an actin cable at the leading edge of these wounds. Scale bar represents 20 μm.

Given the protective effect conferred by ectopic dNrf2 and Gadd45 to naive tissue (Figure 4), we tested whether overexpression of either dNrf2 or Gadd45 could further accelerate the rate of wound repair. We saw the converse with ectopic expression of dNrf2 throughout the epithelium prior to wounding (using the GAL4-UAS system), which caused marked delays in wound closure (Figures 5P and 5Q). This is consistent with published work that suggests excessive and long-term activation of Nrf2 can have detrimental effects on tissues and may even induce cellular senescence [34, 35]. However, ectopic expression of Gadd45 caused a small but significant increase in the rate of wound closure (Figures 5R and 5S). It is therefore likely that for best therapeutic exploitation of these cytoprotective pathways in the clinic, it would be necessary to transiently activate just prior to surgery to avoid any long-term negative effects (see Discussion).

### Wounding Activates a Dynamic Cytoprotective Network of Calcium, JNK, Nrf2, and Gadd45 Signaling

**Figure 6:**
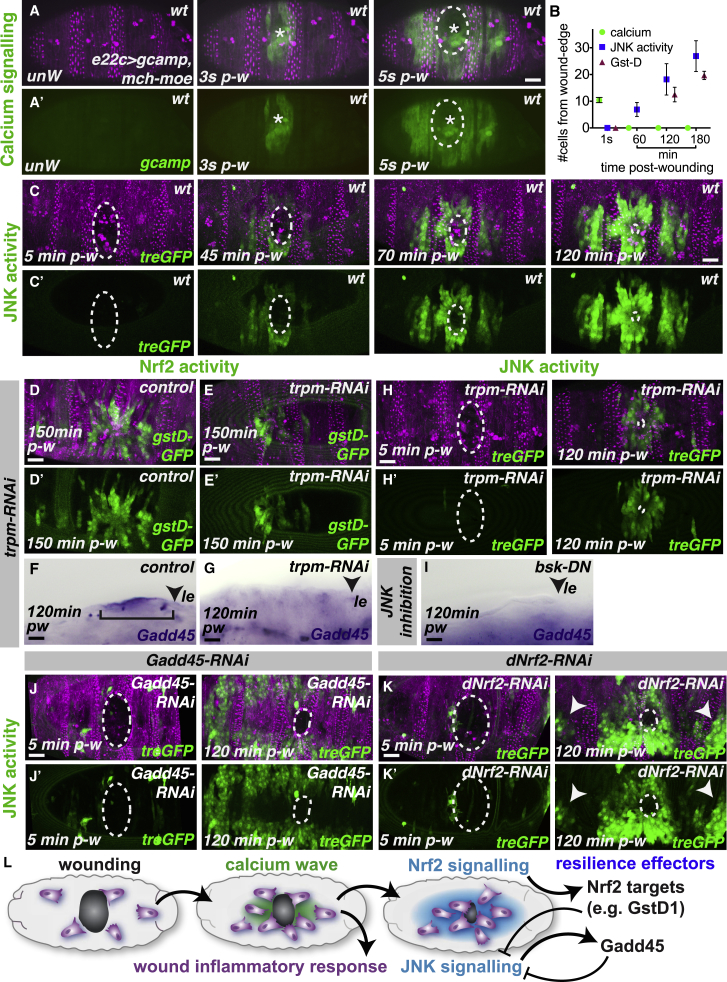
A Complex Network of Wound-Induced Signaling Drives the Expression of Multiple Cytoprotective Factors within the Wounded Epithelium (A–C) Epithelial (magenta Moesin-mCherry, A) wounding triggers a rapid wave of calcium (green, *Gcamp3* reporter, A and A′) through the epithelium spreading up to 10 cell diameters from the wound edge (B). Wounding also activates JNK signaling (green, *tre-GFP* reporter, C) in the surrounding epithelium (magenta, Moesin-mCherry) but with slower dynamics (B and C). (D–H) Inhibition of the wound-induced calcium wave using *trpm-RNAi* causes reduced expression of the Nrf2 target GstD1 (green, E compared to control in D), loss of Gadd45 expression (purple, *in situ* hybridization; F and G) in the wounded epithelium, and reduced activation of JNK signaling (green, H compared to controls in C). (I–K) Wound-induced *Gadd45* expression is also lost following inhibition of JNK signaling using *bsk-dominant negative* (arrowhead, I). Wound-induced JNK signaling (green, *treGFP* in J and K) is elevated in areas further from the wound site following RNAi-mediated inhibition of either *Gadd45* (J) or *dNrf2* (K) compared to controls (C). (L) Schematic illustrates cascading and cross-regulatory network of wound-induced signaling that leads to the upregulation of multiple cytoprotective pathways within the wounded epithelium. Pw, post-wounding; le, leading edge. Scale bars represent 15 μm in (A), (C), (D)–(F), (J), and (K) and 10 μm in (G)–(I). See also Figure S4 and Video S7.

While it is clear that these cytoprotective genes promote tissue resilience and can aid efficient wound repair, what triggers their activation downstream of wounding? The spatial pattern of cytoprotection closely resembles that of the wound-induced calcium wave that spreads out from the injury site within seconds of wounding (Figures 6A and 6B), which we have previously shown to drive inflammatory cell attraction to the wound [17]. We find that JNK signaling is also activated in a similar (but delayed) wave-like pattern at sites of wounding (Figures 6B and 6C; Video S7) using a transgenic reporter of JNK activity (*tre-GFP*) [23], which precedes the wave of Nrf2 reporter activity by approximately 30 min (Figure S4A).

Video S7. Injury Triggers JNK Activation within the Repairing Epithelium, Related to Figure 6 *in vivo* time-lapse imaging of JNK activity within the repairing epithelium of *Drosophila* embryos. JNK activity (green, *tre-GFP* reporter) is undetectable within the unwounded (ventral) epithelium but increases following wounding within the repairing epithelium (magenta, labeled within Moesin-mCherry), in a similar manner to that observed for Nrf2 activity and GstD1 expression, spreading out from the wound edge in a striking wave-like manner, with time-points every 2min. Scale bars represent 10 μm.

Given their similar activation patterns, we tested whether wound-induced calcium or JNK signaling is important for cytoprotective pathway induction (Figures 6D–6I). Previous work has demonstrated that the *Drosophila* TRPM channel is required for efficient propagation of the wound-induced calcium wave, and RNAi-mediated knockdown of TRPM effectively blocks calcium-mediated inflammatory cell recruitment [17]. Using a similar *trpm-RNAi* approach, we found that inhibition of the wound-induced calcium wave significantly reduced Nrf2 activity (as detected using the ARE-GFP reporter; Figures S4B and S4C) as well as expression of the Nrf2 target GstD1 (Figures 6D and 6E) and Gadd45 (Figures 6F and 6G). Strikingly, this suggests that the induction of epithelial resilience is tightly linked to the pathways that initiate the inflammatory response, suggesting the evolution of a “fail-safe” mechanism for tissue protection at any time or site where inflammation is triggered.

In fact, the upregulation of Gadd45 within the wounded epithelium of both *Drosophila* and murine skin requires input from inflammatory cells, as mutants in both species lacking innate immune cells fail to transcriptionally upregulate Gadd45 at the injury site [27] (Figures S4D and S4E). However, Nrf2 and JNK activation appear to be independent of inflammatory cells as both the GstD1 and JNK reporters were upregulated in a similar spatiotemporal pattern to that of control wounds in *srp* mutants (Figures S4F–S4I). While all cytoprotective pathways are thus activated downstream of the initial (calcium) cue that drives inflammation, some resilience machinery (e.g., Gadd45) also requires input from inflammatory cells themselves.

JNK signaling is activated at wounds in both flies and vertebrates [36, 37], where it stimulates the transcription of genes required for wound closure (such as the actin-binding protein Profilin) [38]. Consistent with this, we find that *Drosophila* wounds completely lacking normal JNK activity (using the dominant-negative JNK, *bsk-DN*) exhibit a marked defect in repair and the wounds remain open for many hours (Figures S4J–S4L). Interestingly, we find that full propagation of wound-induced JNK signaling requires the wound-induced calcium wave as *trpm-RNAi* caused a reduction in the spread of JNK activity (Figure 6H) compared to controls (Figure 6C). Given that wound-induced calcium is known to trigger H_2_O_2_ production by the NADPH oxidase Duox [17] and that JNK is redox-sensitive [39, 40], we envision that wound-induced JNK signaling could be amplified by epithelial ROS.

JNK signaling has also been linked to the induction of Gadd45 [28], so we tested whether JNK inhibition affected Gadd45 levels post-wounding; JNK inhibition (again using the dominant-negative JNK, *bsk-DN*) also reduced Gadd45 levels in the wounded epithelium (Figure 6I; compared to control, Figure 6F). Full wound induction of Gadd45 thus appears to require signals originating from both within the repairing epithelium (calcium and JNK) and incoming inflammatory cells.

Intriguingly, vertebrate Gadd45β has been implicated in modulating JNK signaling (e.g., in murine hepatocytes [41]), leading us to speculate that wound-induced Gadd45 could also feed back on wound-associated JNK signaling. To test this, we analyzed JNK signaling following *Gadd45-RNAi* and found that JNK activity was markedly upregulated (Figure 6J; compared to control, Figure 6C). Given the redox-sensitive nature of JNK signaling [39], we hypothesized that Nrf2 signaling could also restrain JNK activity via its role in ROS detoxification; indeed, loss of *dNrf2* led to ectopic activation of JNK signaling at distances further from the wound site (Figure 6K). Remarkably, this elevated JNK activity was also associated with an increase in Gadd45 expression in areas of the epithelium that normally lack Gadd45 expression (Figures S4O and S4P), consistent with the JNK-dependent induction of Gadd45. To confirm the ROS dependence of JNK activation, we tested whether JNK levels were reduced following the overexpression of ROS scavengers (Catalase; Figure S4M) or when ROS production was inhibited (following Duox-RNAi; Figure S4N); in both cases, JNK activity post-wounding was reduced (Figures S4M and S4N) compared to controls (Figure S4J). Given that sustained and excessive levels of JNK signaling have been linked to apoptosis [42] and could be detrimental to repair, Gadd45 and Nrf2 appear to act together to constrain JNK activity so that it remains at safe pro-regenerative levels within the repairing epithelium (schematic, Figure 6L).

## Discussion

Until now, research on cytoprotective factors in wound repair has mainly focused on how antioxidant systems directly minimize ROS-induced damage following injury. However, tissues will undoubtedly have evolved a diverse range of “resilience” mechanisms acting on different cellular targets and working in a highly coordinated manner to collectively reduce damage. In this study, we show that injury activates a cytoprotective signaling network that targets multiple different components to protect the repairing epithelial tissue, including both the upregulation of antioxidant defense machinery and DNA repair mechanisms. In this way, tissue resilience mechanisms can both shield the tissue from damage by directly dampening ROS levels and enhance DNA repair mechanisms (thus making wounded tissues more tolerant to any DNA damage caused by residual ROS). The presence of multiple, partially redundant protective mechanisms ensures effective resilience and thus minimizes delays in tissue repair; indeed, we find that simultaneous knockdown of Nrf2 and Gadd45 exaggerates wound repair defects compared to individual knockouts alone.

Since both Nrf2 and Gadd45α are upregulated within mammalian skin wounds [21, 27], similar networks of wound-induced resilience mechanisms are likely to be well conserved from flies to man. *Drosophila*, with its advanced genetic tractability, capacity for live-imaging, and opportunity for large-scale genetic screening, thus offers an exciting new model for dissecting the mechanisms governing tissue resilience to stress, particularly those during wound repair. Our studies may also have important implications for cancer therapy, as cancer cells could hijack this resilience machinery to protect the tumor from host immune attack, as well as confer resistance to clinical therapies such as chemo- or radiotherapy. Indeed, it is known that Gadd45α deficiency sensitizes epithelial cancer cells to ionizing radiation *in vivo* [43], implicating cytoprotective genes such as Gadd45a as potential drug targets in management of cancer radiotherapy treatments.

For nearly 30 years, experimental biologists and clinicians have observed the remarkable but mysterious phenomenon of “preconditioning,” whereby a brief period of sub-lethal tissue damage triggers adaptive mechanisms that confer subsequent cytoprotection against further insult, either within the same tissue or more remotely [44]. Indeed, recent work in zebrafish has shown that superficial insult (via thoracotomy) preconditions adjacent cardiac tissue and renders it more resilient to subsequent cryoinjury (modeling an infarct) by upregulation of cardioprotective factors [45, 46]. Remarkably, activation of cardioprotective signaling by injection of exogenous ciliary neurotrophic factor just prior to ventricular cryoinjury had beneficial regenerative effects and rendered the heart more resilient to injury [45]. In this regard, therapeutic activation of some or all of these resilience pathways could offer exciting “pre-conditioning” strategies in the clinic to protect patient tissues during surgery or following organ transplant [47].

A better understanding of resilience pathways and their long-term effects (including an analysis of “cost”) is clearly crucial for their full application in a clinical setting, given that excessive and long-term activation of resilience machinery could potentially have adverse effects. Indeed, while we found that ectopic expression of Gadd45 prior to wounding could accelerate wound repair, long-term overexpression of dNrf2 within the epithelium caused marked delays in wound closure. Previous work suggests that prolonged Nrf2 activation may make cells less “competitive” than their neighbors [48] and can also induce certain skin defects (such as hyperkeratosis) [34] and fibroblast senescence [35]. Given the role for wound-induced ROS in inflammatory cell recruitment [3, 17] and angiogenesis [4], we envision that achieving an optimal transient and balanced activation of this endogenous resilience machinery will be the key to unlocking its enormous therapeutic benefits, conferring valuable stress resilience without reaching levels that might otherwise be *detrimental* to repair or later tissue health.

## STAR★Methods

### Key Resources Table

**Table:** 

REAGENT or RESOURCE	SOURCE	IDENTIFIER
**Antibodies**		

anti-γH2AvD	GeneTex	RRID: AB_11165216
anti-8-oxo-dG	Trevigen	RRID: AB_1857195
anti-polyADPribose	BD Biosciences	RRID: AB_394263
anti-GFP	Abcam	RRID: AB_304896
anti-RFP	MBL	RRID: AB_591278
Streptavidin-Cy3	Jackson Immunoresearch	RRID: AB_2337244
Biotinylated anti-mouse	Vector labs	RRID: AB_2687893
Biotinylated anti-rabbit	Vector labs	RRID: AB_2336201
Anti-DIG AP-conjugated antibody	Sigma-Aldrich	RRID: AB_2734716

**Bacterial and Virus Strains**		

Library Efficiency DH5α Competent Cells	Invitrogen	#18263012

**Chemicals, Peptides, and Recombinant Proteins**		

Vectashield	Vector labs	RRID: AB_2336789
Heptane	Sigma	#246654
Formaldehyde 37%	Sigma	# 47608
Methanol	Sigma	# 34860
Hydrogen peroxide	Sigma	#95321
Triton-X	Sigma	#X-100
Tween-20	Sigma	# P9416
Trizol	Invitrogen	#15596026
10S Voltalef oil	VWR	#24627.188
DHE	Invitrogen, Molecular Probes	#D11347
H2DCF	Invitrogen, Molecular Probes	#D399
PBS	Sigma	#P5493
Bovine serum albumin	Sigma	#A3983
Proteinase K	Invitrogen	#25530049
Glycine	Sigma	#410225
Denhardts solution	Invitrogen	#750018
NBT	Roche	#11383213001
BCIP	Roche	#11383221001
Durcupan	Sigma	#44610

**Critical Commercial Assays**		

RNeasy Mini Kit	QIAGEN	# 74104
Thermo Scientific Maxima First Strand cDNA Synthesis Kit	Thermo Scientific	# K1641
PowerUp SYBR Green Supermix	Applied Biosystems	#A25741

**Experimental Models: Organisms/Strains**		

*Drosophila*: *ubiquitous-moesin-GFP*	[49]	N/A
*Drosophila*: *serpent-Gal4*	[50]	N/A
*Drosophila*: *UAS-GFP*	Bloomington Drosophila Stock Center	RRID: BDSC_6874
*Drosophila*: *dEcadherin-GFP*	Kyoto Stock Center	#109007
*Drosophila*: *His2Av-mRFP1*	Bloomington Drosophila Stock Center	RRID: BDSC_23651
*Drosophila*: *ARE-GFP*	[23]	N/A
*Drosophila*: *GstD-ARE:GFP*	[22]	N/A
*Drosophila*: *da-Gal4*	Bloomington Drosophila Stock Center	RRID: BDSC_55851
*Drosophila*: *UAS-moesin-mCherry*	[2]	N/A
*Drosophila*: *OregonR*	Bloomington Drosophila Stock Center	RRID: BDSC_2376
*Drosophila*: *UAS-Gadd45*	[26]	N/A
*Drosophila*: *UAS-dNrf2*	[22]	N/A
*Drosophila*: *UAS-Gadd45-RNAi*	Bloomington Drosophila Stock Center	RRID: BDSC_35023
*Drosophila*: *UAS-dNrf2-RNAi*	[22]	N/A
*Drosophila*: *UAS-dNrf2-RNAi*	Bloomington Drosophila Stock Center	RRID: BDSC_40854
*Drosophila*: *e22c-Gal4*	Bloomington Drosophila Stock Center	RRID: BDSC_1973
*Drosophila*: *UAS-gcamp3*	[17]	N/A
*Drosophila*: *TRE-GFP*	[23]	N/A
*Drosophila*: *UAS-catalase*	Bloomington Drosophila Stock Center	RRID: BDSC_24621
*Drosophila*: *UAS-Duox-RNAi*	[51]	N/A
*Drosophila*: *UAS-trpm-RNAi*	Bloomington Drosophila Stock Center	RRID: BDSC_31672
*Drosophila*: *UAS-basket-DN*	Bloomington Drosophila Stock Center	RRID: BDSC_6409
*Drosophila*: *srp*^*3*^	[27]	N/A
*Drosophila*: *srp*^*AS*^	[27]	N/A
*Drosophila*: *UAS-reaper*	Bloomington Drosophila Stock Center	RRID: BDSC_5824

**Oligonucleotides**		

dNrf2 F-primer CTGCATCGTCATGTCTTCCAGT	Eurofins Genomics	N/A
dNrf2 R-primer AGCAAGTAGACGGAGCCAT	Eurofins Genomics	N/A
Gadd45 F-primer GGTACTGCTGGAGGCCTTTT	Eurofins Genomics	N/A
Gadd45 R-primer CGCAGTAGTCGACTAGCTGG	Eurofins Genomics	N/A
Rpl32 F-primer AGCATACAGGCCCAAGATCG	Eurofins Genomics	N/A
Rpl32 R-primer TGTTGTCGATACCCTTGGGC	Eurofins Genomics	N/A

**Recombinant DNA**		

RE38191 cDNA clone	BDGP	# FBcl0207762

**Software and Algorithms**		

GraphPad Prism V6.01	GraphPad Software	https://www.graphpad.com/scientific-software/prism/
ImageJ/Fiji	National Institutes of Health	https://imagej.nih.gov/ij/
Volocity	PerkinElmer	https://www.perkinelmer.com/lab-products-and-services/resources/cellular-imaging-software-downloads.html
Photoshop	Adobe	https://www.adobe.com/uk/products/photoshop.html
Illustrator	Adobe	https://www.adobe.com/uk/products/illustrator.html

### Lead Contact And Materials Availability

Further information and requests for resources and reagents should be directed to and will be fulfilled by the Lead Contact, Helen Weavers (helen.weavers@bristol.ac.uk). This study did not generate new unique reagents.

### Experimental Model and Subject Details

#### *Drosophila* Stocks and Genetics

Fly stocks were maintained according to standard protocols [52]. All crosses were performed at 25**°**C unless otherwise stated. The following *Drosophila* stocks were used: *ubiquitous-moesin-GFP* [49], *serpent-Gal4* (*Drosophila* macrophage (hemocyte) specific driver) [50], *UAS-GFP, dEcadherin-GFP, His2Av-mRFP1 (BL23651), ARE-GFP* [23] (4XARE:GFP-16, Nrf2 activity reporter, gift from Ioannis Trougakos), *GstD-ARE:GFP* [22] (ARE of the gstD gene, gift from Ioannis Trougakos), *da-Gal4, UAS-moesin-mCherry* [2], *OregonR, UAS-Gadd45* (gift from Uri Abdu) [26], *UAS-dNrf2* [22] (gift from Ioannis Trougakos), *UAS-Gadd45-RNAi* (TRiP.HMS01436), *UAS-dNrf2-RNAi* [22] (gift from Ioannis Trougakos), *UAS-dNrf2-RNAi* (TRiP.HMS02021), *e22c-Gal4, UAS-gcamp3* [17], *TRE-GFP* (JNK activity reporter, gift from JP Vincent) [23], *UAS-catalase, UAS-Duox-RNAi* [51], *UAS-trpm-RNAi* (TRiP.JF01465), *UAS-basket-DN, srp*^*3*^*, srp*^*AS*^ and *UAS-reaper*. *Drosophila* mutants and transgenic lines were obtained from the Bloomington Stock Centre unless otherwise stated.

### Method Details

#### Microscopy and Wounding

Embryos of the appropriate developmental stage were collected from overnight apple juice plates, dechorionated in bleach for 1 min and mounted on double-sided sticky tape on glass slides in 10S Voltalef oil (VWR). Wounds were induced using a nitrogen-pumped Micropoint ablation laser tuned to 435nm (Andor Technologies) [17]. For ROS detection, dechorionated embryos were microinjected with DHE (Invitrogen, Molecular Probes) or H2DCF (Invitrogen, Molecular Probes) in PBS and then either wounded (as above) or left unwounded for the equivalent time period to be time-matched controls, before mounting and imaging. Microinjections and UV-induced apoptosis were performed as described previously [2, 15]. Prior to microinjection, mounted embryos were dehydrated at room temperature for 5 min prior to covering with oil. Targeted UV exposure was performed using the 405nm laser on the Leica TCS SP5 confocal microscope and the standard integrated FRAP software with continuous bidirectional scanning at 700Hz for 30-180 scans (120 scans used as standard but dose response experiment utilized a range of different scan lengths as detailed in the graphical representation of data). Imaging was performed on a PerkinElmer UltraView spinning disc system or Leica TCS SP5 confocal microscope. Image processing was performed using ImageJ (NIH), Adobe Photoshop or Adobe Illustrator software. For quantification of % area of oxidative and DNA damage, all processing was performed in ImageJ; briefly, confocal images were converted to binary format and thresholded before using the Analyze/Measure tool to calculate the % area.

#### Immunostaining and *In Situ* Hybridization

Immunostaining was performed using standard techniques with the following antibodies: anti-γH2AvD (rabbit, GeneTex, 1:500), anti-8-oxo-dG (mouse, Trevigen, 1:200), anti-polyADPribose (mouse, LP96-10,1:200), anti-GFP (goat, Abcam, 1:500) and anti-RFP (Rabbit, MBL, 1:500). As for live-imaging experiments, embryos were collected from apple juice plates and dechorionated in bleach. After rinsing with distilled water, dechorionated embryos were fixed for 20 min in a 1:1 heptane and 4% paraformaldehyde (in a phosphate buffer) solution. Following fixation, embryos were devitillinised in 1:1 heptane and methanol by 30 s of vigorous shaking. Embryos were finally rinsed at least three times in methanol and stored at −20**°**C in fresh methanol until required. Fixed embryos were then blocked in phosphate buffered saline with 0.3% Triton-X detergent and 0.5% bovine serum albumin for one h (PBS-TX-BSA). Embryos were then incubated with diluted primary antibody (at appropriate concentrations) in PBS-TX-BSA overnight at 4**°**C. The following day, the primary antibody solution was removed and embryos washed three times in PBS-TX-BSA for a total of 30 min before incubation with the appropriate secondary antibody (diluted at 1:200 in PBS-TX-BSA) for one h at room temperature. An extra amplification step was performed where required using biotinylated secondary antibodies and Streptavidin-conjugated fluorophores. Carefully staged embryos were oriented and mounted on a glass slide in Vectashield and imaging was performed on a Leica SP5 confocal microscope. For H_2_O_2_ treatment, dechorionated embryos were first shaken with a 1:1 mixture of heptane and 100mM H_2_O_2_ in PBS (or PBS only for controls) for 20min prior to fixation and immunostaining. *Gadd45* RNA localization was performed by *in situ* hybridization using DIG-labeled RNA probes generated by *in vitro* transcription from cDNA templates (RE38191, BDGP). Hybridization and staining was performed according to standard protocols [53]. Fixed embryos were rehydrated in 4% formaldehyde for 30 min, prior to Proteinase K treatment for 2 min (2 μL of a 20mg/mL stock) and 2 brief washes in Glycine (2mg/mL solution) all in PBT buffer (1xPBS and 0.1% Tween20). Embryos were again incubated in 4% formaldehyde for 20 min before transfer to Hybridization buffer containing the appropriate *in situ* probe for overnight incubation at 55**°**C. The following day, embryos were removed from the hybridization buffer and washed in PBT before a 2 h incubation in anti-DIG AP-conjugated antibody (1/2000 in PBT). After brief rinses in PBT, staining was developed using 4.5 μL NBT and 3.5 μL X-phosphate (BCIP) in 1ml of alkaline phosphatase buffer (100mM NaCl, 50mM MgCl_2_ and 100mM Tris pH 9.5). Once staining had developed the reaction was stopped by washing in fridge cold PBT. Embryos were then dehydrated using an EtOH series and mounted in Durcupan.

#### RNA isolation, reverse transcription and real-time qPCR

RNA was isolated from control *da-Gal4*, *da-Gal4 > UAS-dNrf2-RNAi* and *da-Gal4 > UAS-Gadd45-RNAi* stage 14/15 embryos by crushing in TRIzol (Life Technologies) and RNA purified using a RNeasy Mini Kit (QIAGEN). Equal quantities of RNA were then reverse transcribed using Thermo Scientific Maxima First Strand cDNA Synthesis Kit and genomic DNA eliminated using ds DNase (Thermo Scientific). Relative quantification of gene expression was performed using PowerUp SYBR Green Supermix with a Real-time PCR machine (QuantStudio Applied Biosystems). *dNrf2* and *Gadd45* gene expression were normalized to the expression of the housekeeping reference gene *rpl32* using the ΔΔCt analysis method. The following primers were used in this study: dNrf2 F-primer CTGCATCGTCATGTCTTCCAGT, R-primer AGCAAGTAGACGGAGCCAT, Gadd45 F-primer GGTACTGCTGGAGGCCTTTT, R-primer CGCAGTAGTCGACTAGCTGG and Rpl32 F-primer AGCATACAGGCCCAAGATCG, R-primer TGTTGTCGATACCCTTGGGC.

### Quantification and Statistical Analysis

For quantification of % area of oxidative and DNA damage, all processing was performed in ImageJ; briefly, confocal images were converted to binary format and thresholded before using the Analyze/Measure tool to calculate the % area. All statistical analysis was performed in Prism (Graphpad) as detailed in the legends to each Figure; data represented graphically as mean ± SEM with ^∗^p < 0.05, ^∗∗^p < 0.01 and ^∗∗∗^p < 0.001 via appropriate statistical tests (such as the Mann-Whitney Test, one-way ANOVA followed by Dunn’s multiple comparisons or multiple t tests followed by Holm-Sidak multiple comparisons correction, as described in each Figure Legend).

### Data and Code Availability

This study did not generate any new computer code or algorithms. The raw confocal imaging datasets supporting the current study are available from the corresponding author on request.

## Supplemental Information

Document S1. Figures S1–S4

Document S2. Article plus Supplemental Information
