# Supplementary material for: Injury Activates a Dynamic Cytoprotective Network to Confer Stress Resilience and Drive Repair
Source: Curr Biol. 2019 Nov 18;29(22):3851–3862.e4. doi: 10.1016/j.cub.2019.09.035 (PMC6868510; doi:10.1016/j.cub.2019.09.035)
Supplement: Document S1. Figures S1–S4 [file mmc1.pdf]

Current Biology, Volume *29*

Supplemental Information

Injury Activates a Dynamic Cytoprotective Network to Confer Stress Resilience and Drive Repair

Helen Weavers, Will Wood, and Paul Martin

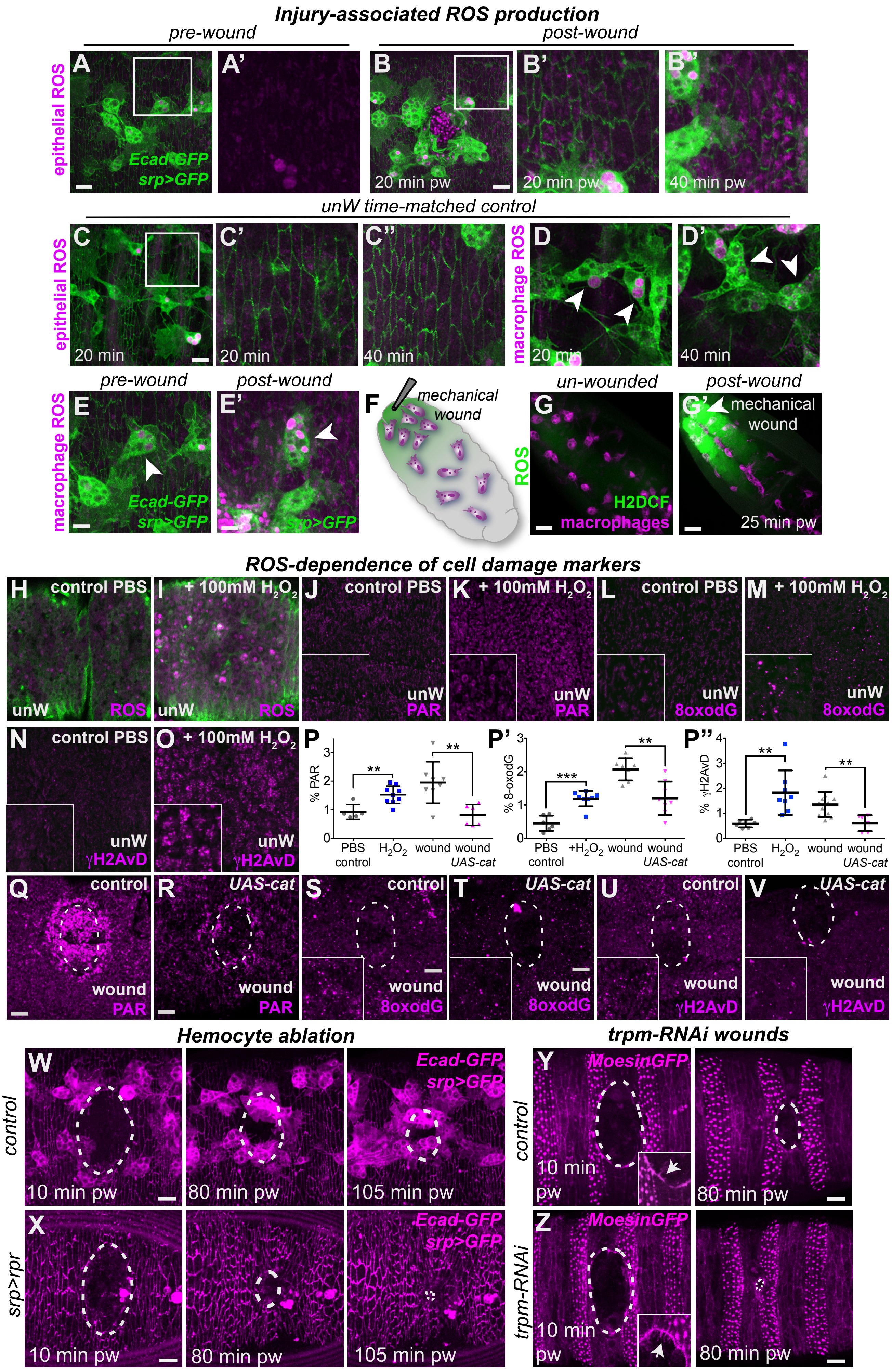
Injury-associated ROS production
pre-wound
post-wound
epithelial ROS
Ecad-GFP
srp>GFP
20 min pw
40 min pw
unW time-matched control
20 min
40 min
macrophage ROS
pre-wound
post-wound
mechanical wound
un-wounded
post-wound
ROS
H2DCF
macrophages
25 min pw
ROS-dependence of cell damage markers
control PBS
+ 100mM H_2O_2
unW
PAR
8oxodG
γH2AvD
% PAR
% 8-oxodG
% γH2AvD
PBS control
H_2O_2
$+H_2O_2$
wound
UAS-cat
control
wound
Hemocyte ablation
trpm-RNAi wounds
srp>rpr
MoesinGFP
trpm-RNAi
10 min pw
80 min pw
105 min pw

Figure S1. Wounding in *Drosophila* triggers ROS production and the inflammatory response delays healing. Related to Figure 1.

Wounding in *Drosophila* embryos triggers an increase in ROS (magenta, DHE labelling superoxide, A-E) levels within the repairing epithelium (A-B; epithelium labelled with *dE-cadherin-GFP*, green, immune cells ('hemocytes') labelled with *srp*>*GFP*, green) and within immune cells (E). Images in E and E' are different views of the same embryos in panels A and B'', respectively. Elevated H_2O_2 levels (green, H2DCF) are also observed following wounding of *Drosophila* embryos (F-G', hemocytes labelled with *srp*>*GFP*, magenta). Unwounded time-matched controls shown for DHE (C-D) and H2DCF (G) staining to control for oxidative conversion of ROS dyes unrelated to wounding. Validation of the ROS-responsive nature of the DNA damage markers (magenta PAR, 8-oxo-dG and γH2AvD) shows that ROS and DNA damage levels are increased following exposure to H_2O_2 (H-O, quantified in P) compared to controls (PBS alone) whilst they are reduced following expression of the Catalase enzyme (Q-V and quantified in P). Inhibition of inflammation, either by genetic ablation of hemocytes (W-X, *srp*>*reaper*) or *RNAi*-mediated inhibition of *trpm* expression (Y-Z), accelerates wound repair (epithelium labelled using Moesin-mCherry) compared to controls.

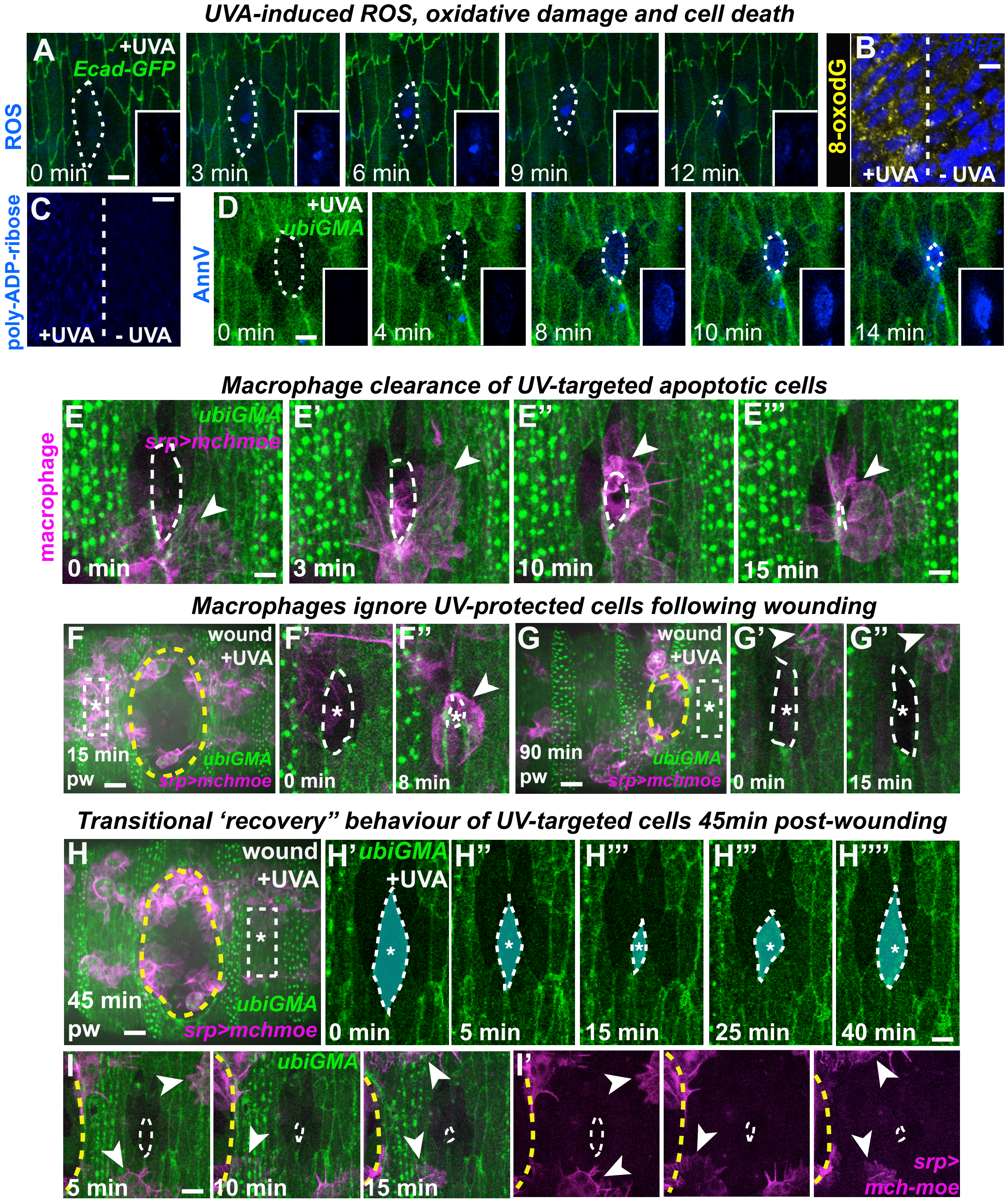

UVA-induced ROS, oxidative damage and cell death
A
+UVA
Ecad-GFP
ROS
0 min
3 min
6 min
9 min
12 min
8-oxodG
B
+UVA
- UVA
C
poly-ADP-ribose
+UVA
- UVA
D
+UVA
ubiGMA
AnnV
0 min
4 min
8 min
10 min
14 min
Macrophage clearance of UV-targeted apoptotic cells
macrophage
E
ubiGMA
srp>mchmoe
0 min
E'
3 min
E''
10 min
E'''
15 min
Macrophages ignore UV-protected cells following wounding
F
wound
+UVA
15 min
pw
ubiGMA
srp>mchmoe
F'
0 min
F''
8 min
G
wound
+UVA
90 min
pw
ubiGMA
srp>mchmoe
G'
0 min
G''
15 min
Transitional 'recovery" behaviour of UV-targeted cells 45min post-wounding
H
wound
+UVA
45 min
pw
ubiGMA
srp>mchmoe
H' ubiGMA
+UVA
0 min
H''
5 min
H'''
15 min
H''''
25 min
H'''''
40 min
I
5 min
ubiGMA
10 min
15 min
I'
srp>
mch-moe

Figure S2. Wounding in *Drosophila* embryos triggers the induction of stress-resistance. Related to Figure 2.

UV-A treatment of naïve epithelial cells causes a dramatic increase in levels of ROS (blue, DHE, A), 8-oxo-dG (yellow, B; dashed line delineates UVA-exposed region, left from non-exposed, right), poly-ADP-ribose (blue, C) and AnnexinV staining (blue, D). Delaminating epithelial cells (green, ubiquitous GFP-tagged Moesin) targeted with UV-A within naïve unwounded embryos are cleared by migrating hemocytes (magenta, *srp>Moesin-mCherry*, E) as are UV-targeted cells which delaminate within the first 15min post-wounding (F). Epithelial cells within the protected zone that fail to delaminate are ignored by nearby hemocytes (G). Epithelial cells targeted within 45min post-wounding show a transitional behaviour and recover after initial rounding up (H) and are ignored by nearby hemocytes (I). Images in (I) are taken from the same embryo as shown in (H). pw, post-wounding. Scale bars represent 5μm in panels A-E, F'-F'', G'-G'' and H'-H'''' and 10μm in panels F, G and H.

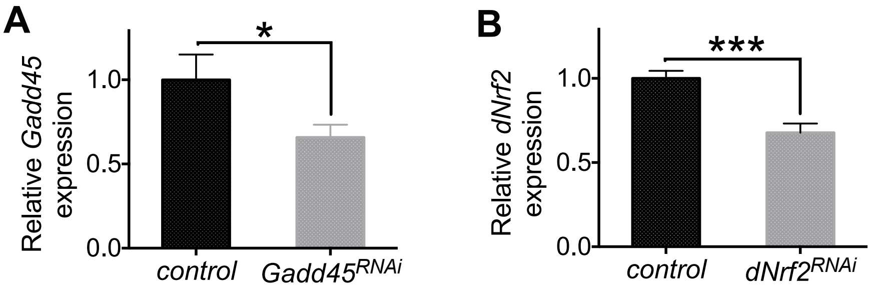
A
Relative Gadd45 expression
1.0
0.5
0.0
*
control
Gadd45^RNAi
B
Relative dNrf2 expression
1.0
0.5
0.0
***
control
dNrf2^RNAi

Figure S3. Nrf2 and Gadd45 RNAi significantly reduce expression levels of their respective targets. Related to Figure 5.

RT-qPCR of the relative expression of *dNrf2* (A) and *Gadd45* (B) within whole stage 14/15 embryos following *Gal4* mediated expression of *dNrf2-RNAi* (A) and *Gadd45-RNAi* (B) within the embryonic epithelium; threshold cycle (Ct) values normalised to Rpl32 reveals a significant reduction in *dNrf2* and *Gadd45* expression.

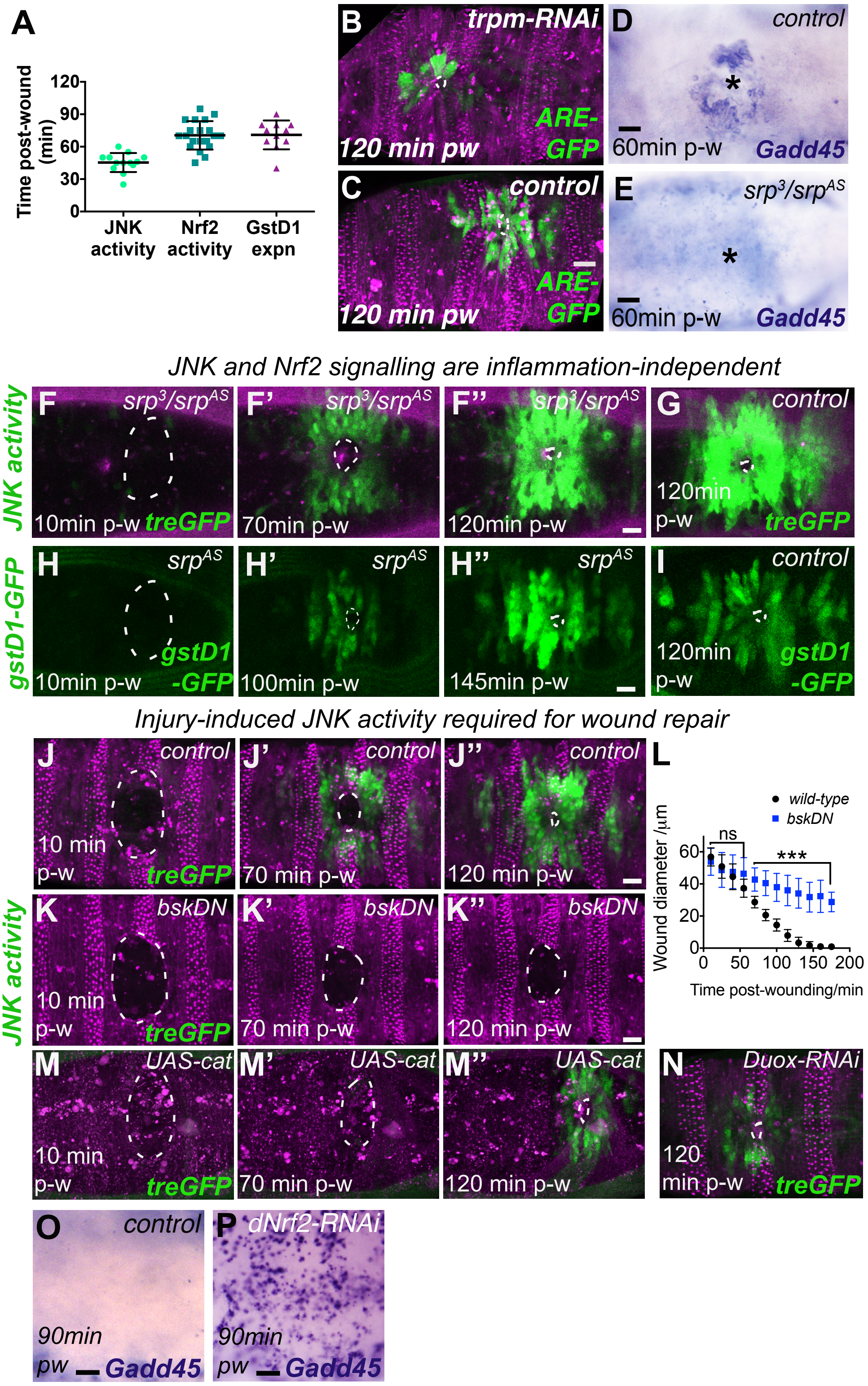

A
Time post-wound (min)
120
90
60
30
0
JNK activity
Nrf2 activity
GstD1 expn
B
trpm-RNAi
ARE-GFP
120 min pw
C
control
ARE-GFP
120 min pw
D
control
60min p-w
Gadd45
E
srp3/srpAS
60min p-w
Gadd45
JNK and Nrf2 signalling are inflammation-independent
JNK activity
F srp3/srpAS
10min p-w treGFP
F' srp3/srpAS
70min p-w
F'' srp3/srpAS
120min p-w
G control
120min p-w
treGFP
gstD1-GFP
H srpAS
gstD1-GFP
10min p-w
H' srpAS
100min p-w
H'' srpAS
145min p-w
I control
120min p-w
gstD1-GFP
Injury-induced JNK activity required for wound repair
J control
10 min p-w
treGFP
J' control
70 min p-w
J'' control
120 min p-w
L
wild-type
bskDN
ns
***
Wound diameter /µm
60
40
20
0
0 50 100 150 200
Time post-wounding/min
JNK activity
K bskDN
10 min p-w
treGFP
K' bskDN
70 min p-w
K'' bskDN
120 min p-w
M UAS-cat
10 min p-w
treGFP
M' UAS-cat
70 min p-w
M'' UAS-cat
120 min p-w
N Duox-RNAi
120 min p-w
treGFP
O control
90min pw
Gadd45
P dNrf2-RNAi
90min pw
Gadd45

Figure S4. A dynamic network of wound-induced signalling pathways drive tissue resilience. Related to Figure 6.

JNK signalling is activated in response to wounding (A, time at which reporter activity first detected within repairing epithelium), prior to that observed for Nrf2 signalling and GstD1 expression. *Trpm-RNAi* mediated inhibition of the inflammatory calcium wave reduced activation of the Nrf2 activity reporter *ARE-GFP* (green, B-C) around the wound site (epithelium magenta, Moesin-mCherry). Wound-induced expression of *Gadd45* (control, D) is reduced in *srp* mutant embryos that lack a wound-induced inflammatory response (E). Inflammation *per se* is not required for wound-induced activation of JNK (green, *tre-GFP*, F-G) or GstD1 expression (green, *gstD-GFP* reporter, H-I) as JNK and GstD1 reporter activity resembles that of control embryos (G and I, respectively) in *srp* mutants. JNK signalling (green, *tre-GFP*, J), activated in response to wounding, is required for efficient wound repair in *Drosophila* embryos as inhibition of JNK signalling using *UAS-basket-dominant-negative* (K) causes a significant delay in wound closure (K and quantified in L). JNK signalling is responsive to ROS levels as expression of Catalase (M) or Duox-RNAi (N) reduced the activation of the *treGFP* JNK reporter. RNAi-mediated inhibition of dNrf2 expression caused elevated Gadd45 expression (P) in regions of the epithelium that normally lack Gadd45 (O). pw, post-wounding. Data in represented as mean ± SEM; $*p < 0.05$, $**p < 0.01$, $***p < 0.001$ via the multiple t-tests followed by Holm-Sidak multiple comparisons test (L). Scale bars represent 10μm in panels B-P.
