# Supplementary material for: Injury Activates a Dynamic Cytoprotective Network to Confer Stress Resilience and Drive Repair
Source: Curr Biol. 2019 Nov 18;29(22):3851–3862.e4. doi: 10.1016/j.cub.2019.09.035 (PMC6868510; doi:10.1016/j.cub.2019.09.035)
Supplement: Document S2. Article plus Supplemental Information [file mmc9.pdf]

Article

Current Biology

Injury Activates a Dynamic Cytoprotective Network to Confer Stress Resilience and Drive Repair

Graphical Abstract

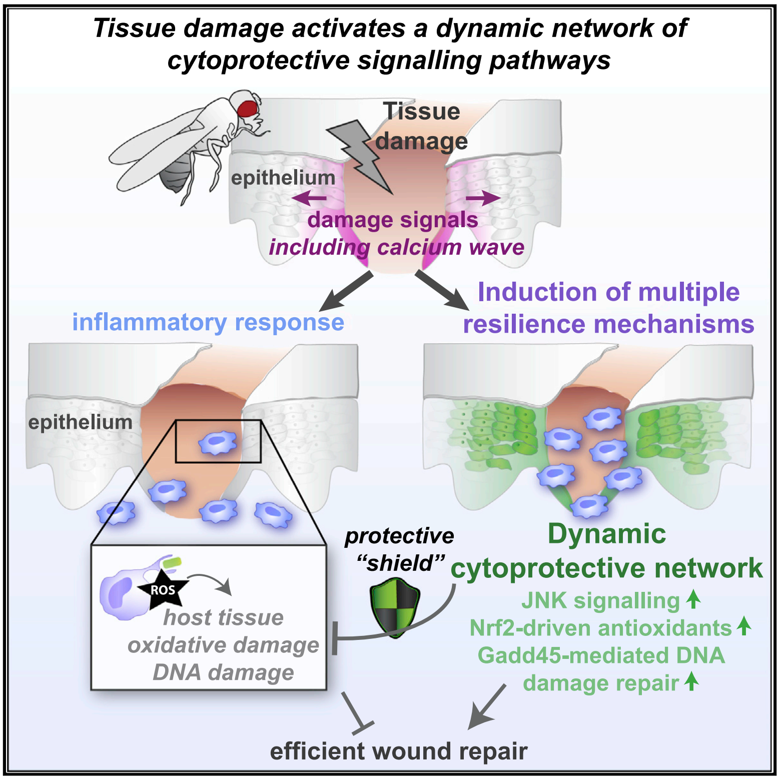

Authors

Helen Weavers, Will Wood, Paul Martin

Correspondence

helen.weavers@bristol.ac.uk

In Brief

Weavers et al. use live-imaging and genetic approaches in *Drosophila* to identify how tissue damage activates a dynamic cytoprotective network (involving JNK, Nrf2, and Gadd45) within the repairing epithelium, which confers stress "resilience" by protecting against inflammatory ROS damage, and is essential for driving efficient wound repair.

Highlights

- Tissue damage and inflammation activate cytoprotective genes to confer resilience
- Nrf2 and Gadd45 resilience factors are required for effective wound repair *in vivo*
- Nrf2 and Gaddd45 limit inflammatory ROS damage and promote DNA damage repair
- Ectopic resilience gene induction can protect naive unwounded tissue from damage

Weavers et al., 2019, Current Biology *29*, 3851–3862
November 18, 2019 © 2019 The Author(s). Published by Elsevier Ltd.
https://doi.org/10.1016/j.cub.2019.09.035

Current Biology
Article

CellPress

Injury Activates a Dynamic Cytoprotective Network to Confer Stress Resilience and Drive Repair

Helen Weavers,[1,7,*] Will Wood,[2,3,6] and Paul Martin[1,4,5,6]
[1]School of Biochemistry, Biomedical Sciences, University of Bristol, Bristol BS8 1TD, UK
[2]School of Cellular and Molecular Medicine, Biomedical Sciences, University of Bristol, Bristol BS8 1TD, UK
[3]Centre for Inflammation Research, University of Edinburgh, Queens Medical Research Institute, Edinburgh EH16 4TJ, UK
[4]School of Physiology, Pharmacology and Neuroscience, Biomedical Sciences, University of Bristol, Bristol BS8 1TD, UK
[5]School of Medicine, Cardiff University, Cardiff CF14 4XN, UK
[6]These authors contributed equally
[7]Lead Contact
*Correspondence: helen.weavers@bristol.ac.uk
https://doi.org/10.1016/j.cub.2019.09.035

SUMMARY

In healthy individuals, injured tissues rapidly repair themselves following damage. Within a healing skin wound, recruited inflammatory cells release a multitude of bacteriocidal factors, including reactive oxygen species (ROS), to eliminate invading pathogens. Paradoxically, while these highly reactive ROS confer resistance to infection, they are also toxic to host tissues and may ultimately delay repair. Repairing tissues have therefore evolved powerful cytoprotective "resilience" machinery to protect against and tolerate this collateral damage. Here, we use *in vivo* time-lapse imaging and genetic manipulation in *Drosophila* to dissect the molecular and cellular mechanisms that drive tissue resilience to wound-induced stress. We identify a dynamic, cross-regulatory network of stress-activated cytoprotective pathways, linking calcium, JNK, Nrf2, and Gadd45, that act to both "shield" tissues from oxidative damage and promote efficient damage repair. Ectopic activation of these pathways confers stress protection to naive tissue, while their inhibition leads to marked delays in wound closure. Strikingly, the induction of cytoprotection is tightly linked to the pathways that initiate the inflammatory response, suggesting evolution of a fail-safe mechanism for tissue protection each time inflammation is triggered. A better understanding of these resilience mechanisms—their identities and precise spatiotemporal regulation—is of major clinical importance for development of therapeutic interventions for all pathologies linked to oxidative stress, including debilitating chronic non-healing wounds.

INTRODUCTION

Reactive oxygen species (ROS) are universal injury-induced signals, produced by NADPH oxidases as an immediate response to tissue damage [1]. At low levels, ROS can function as attractants for the recruitment of innate immune cells [2, 3] and to promote efficient wound angiogenesis [4]; however, incoming inflammatory cells generate additional ROS in a "respiratory burst" to eliminate invading pathogens and confer resistance to infection [5, 6]. Although this bacteriocidal response is clearly beneficial, excessive ROS levels can cause substantial bystander damage to host tissue [5]; indeed, excessive oxidative stress is thought to be a key player in the pathogenesis of chronic non-healing wounds of patients in the clinic [7–9].

To counter inflammatory stress, host tissues must employ powerful cytoprotective machinery to limit the "collateral" damage and prevent immunopathology [10]. Mammalian wound studies have identified a number of signaling pathways that may promote protection against oxidative stress [11, 12], but such investigations have been complicated by the intricacy of the protection machinery and relative genetic intractability of vertebrate models. Nevertheless, a better understanding of these protective mechanisms will be crucial to enable the development of improved therapeutic interventions for a wide range of oxidative stress-related diseases, including chronic non-healing wounds. Also in the context of wound repair, therapeutic activation of cytoprotective pathways in the clinic could also offer an exciting approach to "precondition" patient tissues prior to elective surgery [13].

Here, we develop a novel experimentally amenable *Drosophila* model in which to dissect the complex cytoprotective mechanisms that render repairing tissues "resilient" to inflammation-derived damage. *Drosophila* is a well-established model for uncovering fundamental, conserved aspects of wound repair and the inflammatory response [14–16] and offers unrivalled genetic tractability and optical translucency for high-resolution *in vivo* imaging.

In this study, we characterize the temporal and spatial dynamics of the stress "resilience" mechanisms that are induced downstream of wounding and dissect the underlying molecular and cellular mechanisms driving tissue protection. We identify a complex cross-regulatory network of cytoprotective pathways, involving calcium, JNK, Nrf2, and Gadd45, which collectively "shield" tissues from ROS-induced damage and promote efficient damage repair. RNAi-mediated inhibition of either Nrf2 or Gadd45 delays wound repair, which is further exacerbated if both pathways are inhibited. Interestingly, we find that these cytoprotective pathways are activated downstream of the same

This is an open access article under the CC BY license (http://creativecommons.org/licenses/by/4.0/).

calcium signaling pathway that initiates the inflammatory response, suggesting the existence of a "fail-safe" mechanism for cytoprotection whenever inflammation is triggered. Finally, ectopic activation of these protective pathways can confer stress resilience to naive unwounded tissue, and in the case of Gadd45, can even accelerate the rate of wound repair. Prolonged activation of Nrf2, however, caused marked delays in wound repair, suggesting that the optimal level of cytoprotection required for the most efficient tissue repair will be a finely tuned spatiotemporal balance of cytoprotective signaling.

RESULTS

Tissue Damage Triggers a Burst of Inflammatory ROS and ROS-Induced Damage

A dramatic increase in ROS levels occurs during the inflammatory wound response (Video S1) within *Drosophila* embryos (Figures 1A–1C; higher magnification views, Figures S1A–S1G) [2]; this is accompanied by a significant increase in levels of oxidative DNA damage (base adduct 8-oxo-dG; Figures 1D and 1E; quantified in Figure 1F) and activation of the DNA damage response within epithelial cells at the wound margin (γH2AvD, the *Drosophila* ortholog of mammalian γH2AX; Figures 1G and 1H; quantified in Figure 1I; PARylation, Figures 1J and 1K). These DNA damage markers (PAR, 8-oxo-dG, and γH2AvD) are highly responsive to ROS levels, as shown by exposure to exogenous H_2O_2 or ectopic expression of the antioxidant enzyme Catalase (Figures S1H–S1V). The high levels of ROS and oxidative damage suggest that the wound-induced inflammatory response (despite being necessary to fight potential infection) might also be detrimental to tissue repair. We therefore inhibited wound inflammation, either by genetic ablation of immune cells (termed "hemocytes" in *Drosophila*, using *srp-Gal4*-driven expression of the pro-apoptotic gene *reaper*; Figures 1L, S1W, and S1X) or by blocking propagation of the pro-inflammatory calcium wave [17] (using RNAi-mediated inhibition of the Trpm calcium channel; Figures 1M, S1Y, and S1Z); in both cases, wound closure was accelerated in the absence of inflammation (Figures 1L and 1M). Detailed analysis of wound closure indicates that, while the rapidly assembled actin cables at the wound leading edge appeared indistinguishable from controls (insets, Figures S1Y and S1Z), the repairing epithelial sheet migrated faster than normal to seal the wound—suggesting that inflammatory ROS may normally impede cell migration. Indeed, ROS production was significantly reduced following immune cell ablation (Figures 1N and 1O) compared to controls.

Wounding Induces a Zone of Stress Resilience within the Repairing Epithelium

Given the marked increase in ROS production and oxidative damage following wounding, it is perhaps somewhat surprising that only minimal levels of apoptosis are normally observed around healthy wounds with a standard robust inflammatory response (our previous work) [15]. To explain this, we envision that injured tissues might normally activate protective pathways to counter inflammation-associated damage. To investigate such a phenomenon, we developed a proxy model to test the sensitivity of the wounded epithelium to stress (Figure 2), using micro-irradiation with UV-A light. Individual cells within the "naive" unwounded epithelium of *Drosophila* embryos are highly sensitive to UV-A-induced damage and rapidly undergo apoptosis (Figures 2A–2E; Video S2) [15]. UV-A induced ROS production within the targeted cells (Figures 2C and S2A), and this is associated with an increase in a variety of DNA lesions, including the oxidative base adduct 8-oxo-dG (Figure S2B), poly-ADP-ribose (Figure S2C), and double-strand DNA breaks (γH2AvD; compare Figure 2D with Figure 1G′). Individual UV-damaged cells rapidly delaminate from the epithelium (Figure 2B) while exhibiting positive AnnexinV staining on their surface (Figures 2E and S2D) and are rapidly engulfed by migrating macrophages (Figure S2E) [15]. Such apoptotic stress responses are generally considered critical fail-safe mechanisms to prevent malignant transformation, with excessive unrepaired DNA damage and high levels of ROS leading to activation of death-receptor signaling [18].

Strikingly, we find that the epithelium of wounded embryos develops increased resistance to UV-induced apoptosis in a strict spatiotemporal manner following injury (Figure 2A). Individual epithelial cells in the vicinity of the wound, if targeted with UV-A within the first 30 min post-wounding, display similar sensitivity to those within an unwounded epithelium, rapidly rounding up and delaminating basally (Figure 2F; Video S3), with removal by macrophages (Figure S2F). However, with increasing time post-wounding, these cells display a striking change in UV-A sensitivity (Figures 2G–2J). From 60 min post-wounding onward, cells extending back up to 10 cell diameters from the wound margin within the repairing epithelium become more resistant to the UV-A-induced apoptosis and often fail to delaminate (Figure 2G; quantified in Figure 2H and Video S3). The proportion of cells exhibiting this UV resistance increases until 120 min post-wounding, but the protective effect is temporary, and UV resistance steadily declines from 3 h post-wounding onward (Figure 2H). The response of targeted cells to UV exposure followed a typical "dose-response" relationship, with increased UV exposure times inducing a progressively higher proportion of epithelial cell death for both unwounded and wounded tissues (Figure 2I); nevertheless, cells around the wound edge could resist significantly higher UV doses than cells of unwounded controls (Figure 2I).

The protective effect spreads outward from the wound margin and declines with increasing distance from the wound edge (quantified in Figure 2J) with only minimal protection observed at 20 cells from the wound edge (at 120 min post-wounding) with the majority of cells delaminating after UV exposure (Figure 2K). Epithelial cells targeted with UV within the protected zone that fail to delaminate are also ignored by nearby macrophages (Figure S2G), suggesting the targeted epithelial cells fail to display normal apoptotic "eat me" signals. Intriguingly, cells targeted at an intermediate time point (approximately 45 min post-wounding) display a surprising transitional behavior, initially rounding up (as in unwounded tissues) but then recovering and remaining within the epithelium (Figure S2H), with no associated recognition by nearby macrophages (Figure S2I); it is possible these epithelial cells are able to recover from the "brink of death" similar to that observed recently within certain tissues during *Drosophila* development [19]. These data suggest that cells in the vicinity of repairing epithelial tissues dynamically upregulate protective mechanisms following wounding, which

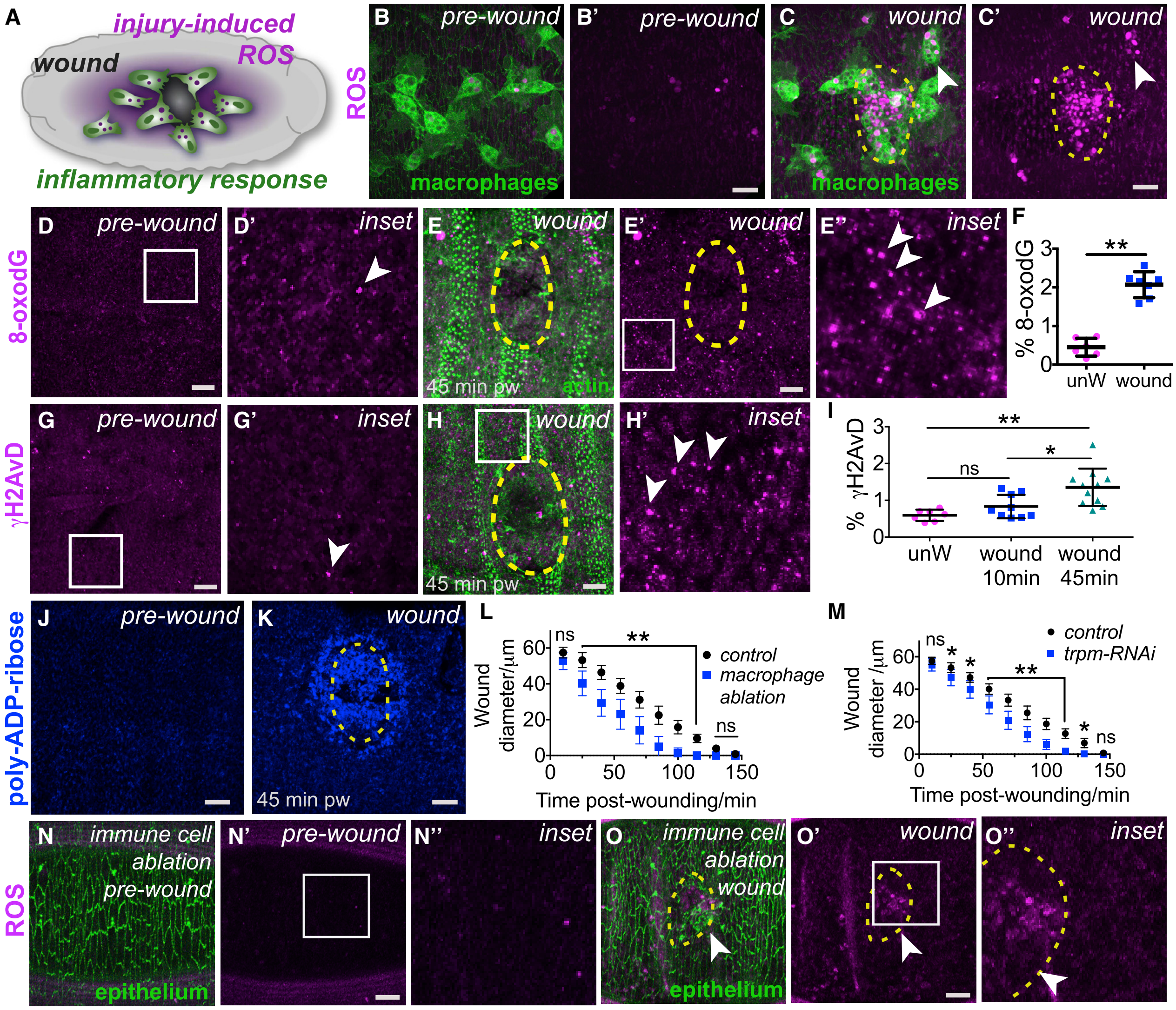

Figure 1. Wound-Induced Inflammation Triggers ROS Production and Oxidative Damage

(A–K) Wounding and inflammation (green immune cells, *srp > GFP*, A–C) in *Drosophila* embryos are associated with increased ROS (magenta, DHE staining) production (A, schematic; B, pre-wound; C, 1 h post-wound; arrowheads indicate ROS within immune cells), oxidative damage (arrowheads, magenta 8-oxo-dG, D and E; quantified in F), γH2AvD puncta (arrowheads, magenta, G and H; quantified in I), and PARylation (blue, J and K). % 8-oxo-dG and % γH2AvD refer to percent (%) of area measured that is positive for marker of interest after thresholding.

(L–O) Inhibition of wound inflammation (macrophage ablation using *srp > reaper* [L] and *trpm-RNAi* [M]) accelerates the rate of wound closure compared to controls (quantified in L and M, n > 20 for each condition). Macrophage ablation is associated with reduced ROS production (magenta DHE staining) before (N) and after (arrowheads, O) wounding compared to controls (B and C).

Wound edge represented by dashed yellow outlines in (C), (E), (H), (K), and (O). Scale bars represent 10 μm in (B)–(E), (G), (H), (J), (K), (N), and (O). Data represented as mean ± SEM; $^{*}p < 0.05$ and $^{**}p < 0.01$ via the Mann-Whitney Test (F), one-way ANOVA followed by Dunn's multiple comparisons test (I), or multiple t tests followed by Holm-Sidak multiple comparisons correction (L and M).

See also Figure S1 and Video S1.

make them more *resilient* to stress-induced cell damage or death.

Wounding Activates Multiple Cytoprotective Pathways

We next investigated which stress-induced pathways could be responsible for driving wound-induced "resilience." The temporal dynamics of protection induction suggest that resilience is likely to, at least in part, require *de novo* transcription or translation. In fact, we find that multiple genes with potential cytoprotective activity are upregulated within the wounded epithelium, with strikingly similar spatiotemporal dynamics to the induction of UV-A resilience (Figure 3A). Nrf2 is a master regulator of the cellular antioxidant response [20] and is transcriptionally activated within mammalian wounds [21]. Using an *in vivo* fluorescence reporter of *Drosophila* Nrf2 activity (Cap "n" collar isoform-C, CncC [22]) [23], we live-imaged the spatiotemporal dynamics of Nrf2 signaling upon wounding (Figure 3B; Video S4) and observed a wave of Nrf2 activity spreading out

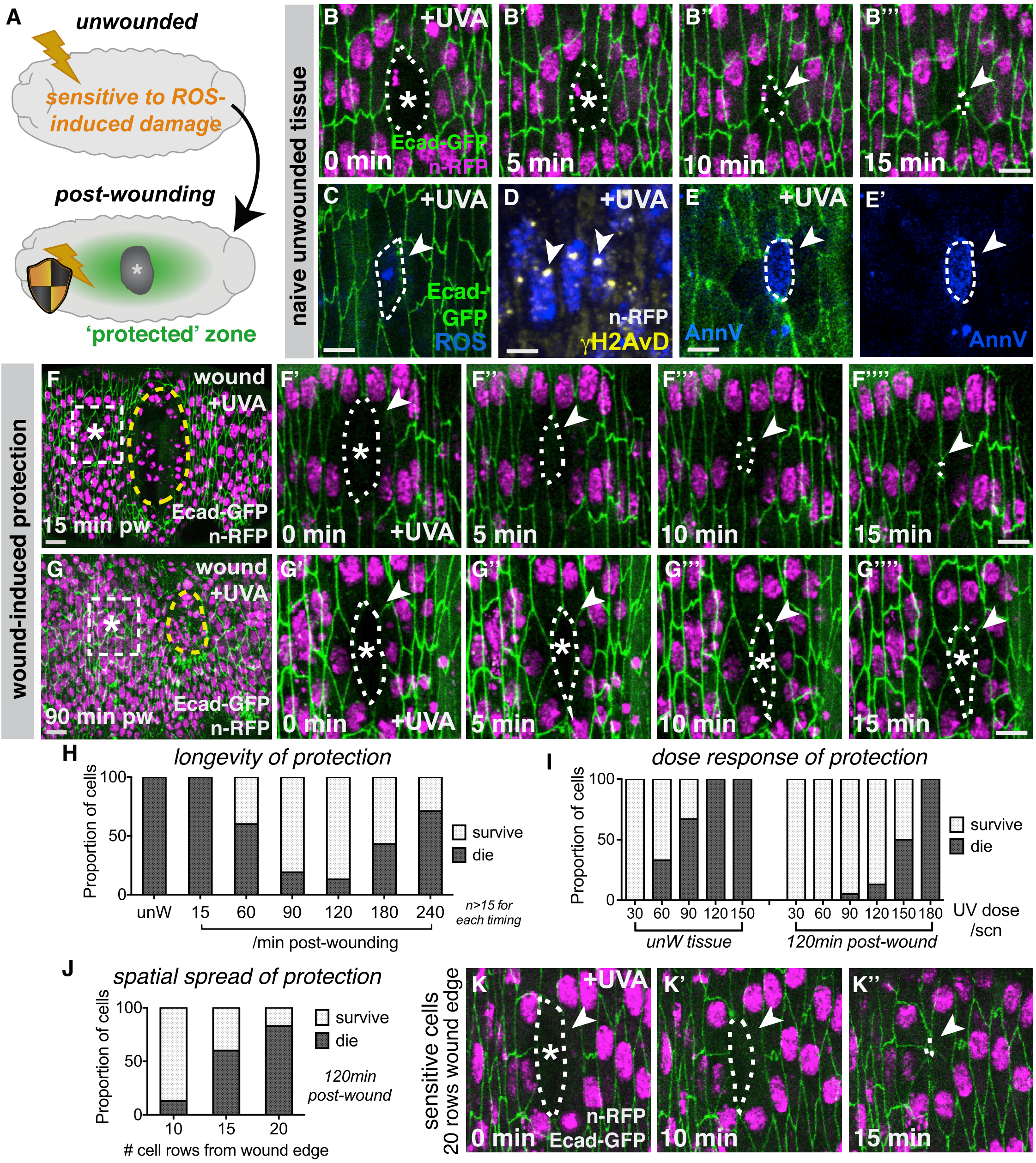

Figure 2. Tissue Damage Activates a Transient Zone of Stress Resilience within the Repairing Epithelium

(A–E) Wounding triggers the epithelium to become more resistant to UV-induced cell damage and death (schematic, A). Naive unwounded tissue is sensitive to UV-A (B–E), with targeted cells (asterisks) rapidly rounding up and delaminating (B; magenta nuclei, *His2Av-mRFP*; green cell outlines, *dE-cadherin-GFP*), with increased ROS (blue DHE in C), γH2AvD puncta (arrowheads, yellow in D), and AnnexinV staining (arrowheads, blue, E and E′).

(F and G) Wounded epithelium (magenta nuclei, *His2Av-mRFP*; green cell outlines, *dE-cadherin-GFP*) initially sensitive to UV-A (F) with targeted cells (asterisks) delaminating from epithelium (arrowheads, F′–F′′′′) as in controls. Wounded epithelium more resistant to UV-induced stress 90 min post-wounding (G) with targeted cells (asterisk) remaining in epithelium (arrowheads, G′–G′′′′).

(H–K) The induction of UV resistance is temporary (quantified in H), displays a typical dose-response behavior (quantified in I), and fades with increasing distance from the wound edge (J and K).

Wound edge represented by dashed yellow outlines in (F) and (G); UV-targeted cells indicated by dashed white line in (B), (C), (E), (F′)–(F′′′′), (G′)–(G′′′′), and (K)–(K′′). pw, post-wounding. Scale bars represent 10 μm in (F) and (G) and 5 μm in (B)–(E), (F′)–(F′′′′), (G′)–(G′′′′), and (K)–(K′′).

See also Figure S2 and Videos S2 and S3.

from the wound margins. As in mammals, *Drosophila* Nrf2 activity can also be regulated at the post-translational level by its binding partner and inhibitor Keap1 [24]. Oxidative stress is known to inactivate Keap1, allowing subsequent Nrf2 stabilization and activation of Nrf2 signaling [25]; a similar oxidative stress-mediated inhibition of Keap1 post-wounding could

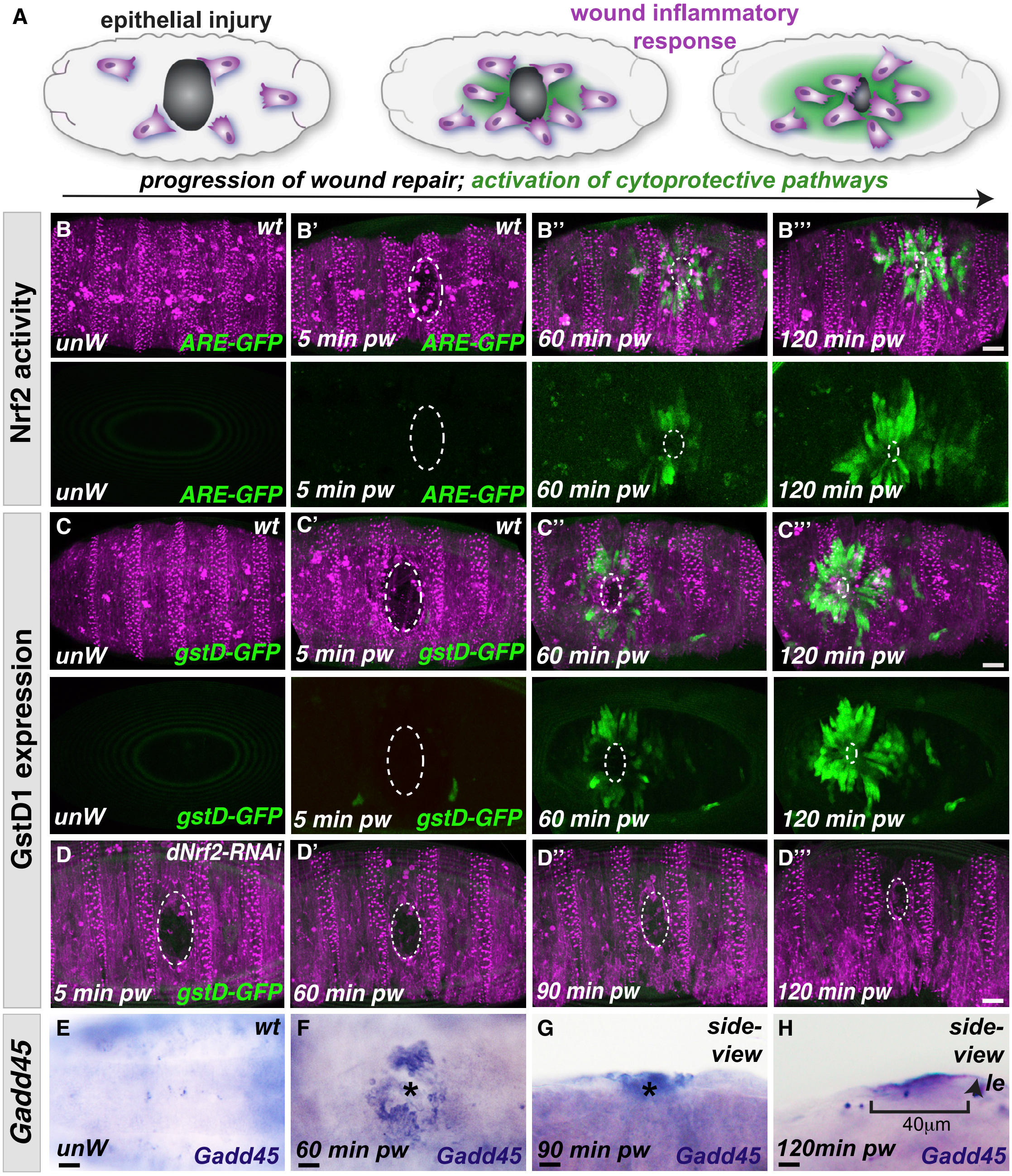

Figure 3. Multiple Cytoprotective Pathways Activated Downstream of Wounding

(A) Epithelial wounding and inflammation (magenta) in *Drosophila* embryos trigger the activation of multiple cytoprotective pathways (green, schematic).

(B) Nrf2 signaling (green, ARE-GFP reporter), which is absent from the epithelium (magenta, ubiquitous Moesin-mCherry) of control unwounded embryos, is activated in a wave spreading out from the wound site.

(C) GstD1 expression (green, gstD-ARE:GFP transgenic reporter) is also absent from control unwounded embryos (magenta epithelium, ubiquitous Moesin-mCherry), but GstD1 expression increases in a similar wave pattern spreading out from the wound.

(D) Wound-induced activation of GstD1 expression (green, *GstD-GFP* reporter) within the repairing epithelium is lost following RNAi-mediated inhibition of *dNrf2* expression compared to controls (C).

(E–H) *Gadd45* expression (purple, *in situ* hybridization, E–H) is also undetectable in control unwounded epithelium (E) but increases in the repairing epithelium following wounding (F, ventral view; G and H, lateral views). *Gadd45* expression extends up to 40 μm back from the wound leading edge (le, arrowhead) within the repairing epithelium 120 min following wounding (H). pw, post-wounding; le, leading edge.

Scale bars represent 15 μm in (B)–(D) and 10 μm in (E)–(G).

See also Videos S4 and S5.

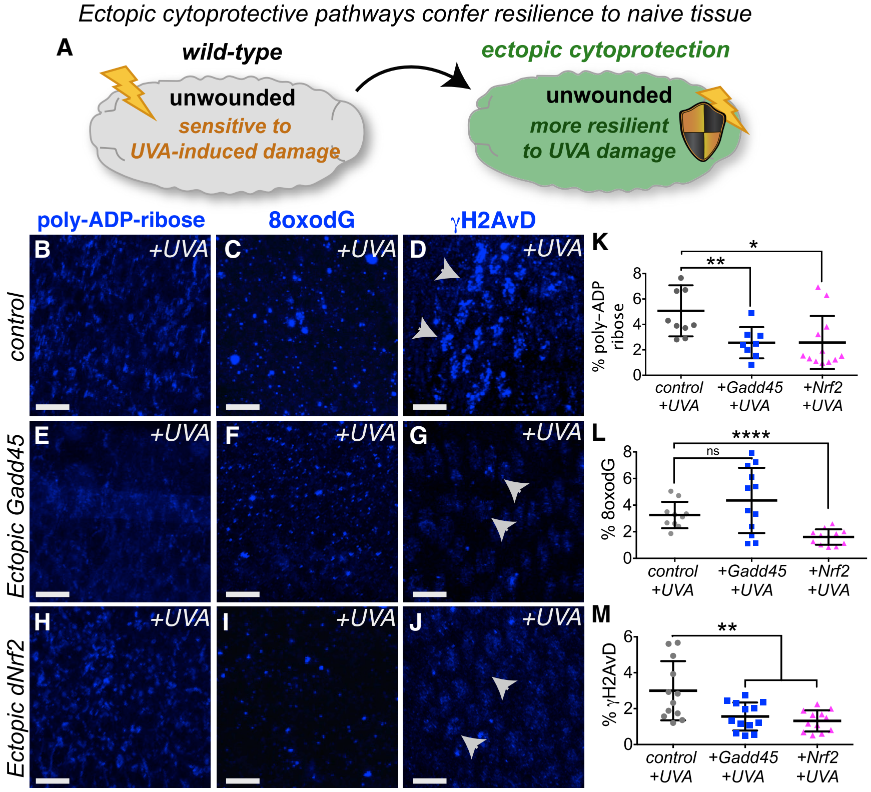

Figure 4. Wound-Induced Pathways Can Confer Protection against Oxidative Damage to Naive Unwounded Tissue

Ectopic expression of either *Gadd45* (A, schematic and E–G) or *dNrf2* (H–J) in unwounded *Drosophila* embryos confers increased protection against UV-induced damage compared to controls (B–D), as shown by poly-ADP-ribose, 8-oxo-dG, and γH2AvD staining (quantified in K–M). Arrowheads (D, G, and J) indicate punctae of γH2AvD staining. % poly-ADP-ribose (PAR), % 8-oxo-dG, and % γH2AvD refer to percent (%) of area measured that is positive for marker of interest after thresholding. Scale bars represent 5 μm in (B)–(J). Data represented as mean ± SEM; ns, not significant, $^*p < 0.05$ and $^{**}p < 0.01$ via multiple t tests followed by Holm-Sidak multiple comparisons correction (K–M).

activate Nrf2 post-translationally in our system. We observe a similar, wound-induced, wave-like expression pattern upon wounding for *Drosophila* GstD1 (Figure 3C; Video S5), a glutathione S transferase (GST) enzyme involved in glutathione-mediated detoxification and a known target of Nrf2 [22]. Consistent with this, we find that *dNrf2-RNAi* expression abolishes the wound-triggered upregulation of *GstD-GFP* (Figure 3D), suggesting that Nrf2 and its downstream targets may confer tissue resilience post-wounding (perhaps via ROS detoxification).

However, we envision that wounded tissues will upregulate a multitude of further protection strategies that target different cellular components and act collectively to reduce damage. Indeed, we find that *Drosophila* Gadd45 (the single fly homolog of the mammalian growth arrest and DNA-damage inducible GADD45 gene family [26]) is transcriptionally induced within the *Drosophila* wounded epithelium with strikingly similar spatiotemporal dynamics (Figures 3E–3H) [27] to Nrf2 activity. Since Gadd45 has been implicated in DNA damage repair in both mammals and flies [28, 29], it could mediate an additional level of protection by promoting repair of DNA damage induced by ROS that escaped Nrf2-mediated detoxification.

Nrf2 and Gadd45 Confer Resilience to Naive Tissues and Are Required for Wound Repair

To determine whether Nrf2 and Gadd45 promote tissue resilience to stress, we tested whether their ectopic activation could confer protection to naive unwounded tissues (Figures 4A–4M). Using the GAL4-UAS system [30] for genetic mis-expression, we find that ectopic expression of *Gadd45* (Figures 4E–4G) or *dNrf2* (Figures 4H–4J) indeed confers stress resilience to cells within the unwounded epithelium of *Drosophila* embryos compared to controls (Figures 4B–4D). Unlike the high levels of DNA damage (as detected by PARylation [Figure 4B] and γH2AvD [Figure 4D]) induced by UV-A irradiation of control cells, cells with elevated Gadd45 expression exhibit remarkable resistance to UV-A-induced DNA damage (Figures 4E and 4G; quantified in Figures 4K and 4M, respectively), although levels of oxidative damage (as detected by 8-oxo-dG) remain indistinguishable from controls (Figures 4C, 4F, and 4L). Epithelial cells with elevated Nrf2 levels also exhibit significantly less damage than control UV-A-irradiated cells (as detected by PARylation, 8-oxo-dG, and γH2AvD; Figures 4H–4J; quantified in Figures 4K–4M). Interestingly, however, ectopic Nrf2 alone wasn't sufficient to completely protect these cells from UV-A-induced death (data not shown), suggesting that full stress protection (as observed upon wounding) requires the activity of multiple cytoprotective pathways.

We next tested whether these resilience pathways are required for efficient wound repair *in vivo* (Figures 5A–5O). RNAi-mediated knockdown of *Drosophila* Nrf2 (Figures 5C–5H; using multiple independent RNAi lines) or Gadd45 (Figures 5I–5M) caused significant delays in wound closure compared to controls (Figure 5B; Video S6), despite initial assembly of a robust actin cable at the wound leading edge (insets, Figures 5B, 5C, and 5I). Detailed analysis indicated that the repairing epithelium failed to migrate as fast as controls, and this was accompanied by a breakdown in the actin cable at the leading edge by 120 min post-wounding (insets, Figures 5B″, 5C″, and 5I″). These repair defects were associated with increased levels of DNA damage (Figures 5F–5H and 5K–5M) when compared to that of control wounds, suggesting that Nrf2 and Gadd45 are normally required to protect the repairing epithelium from damage. Interestingly, previous reports suggest that oxidative stress negatively impacts cell migration and cytoskeletal organization in various cell types [31, 32]. qRT-PCR was performed to validate that the *dNrf2-RNAi* and *Gadd45-RNAi* lines effectively knock

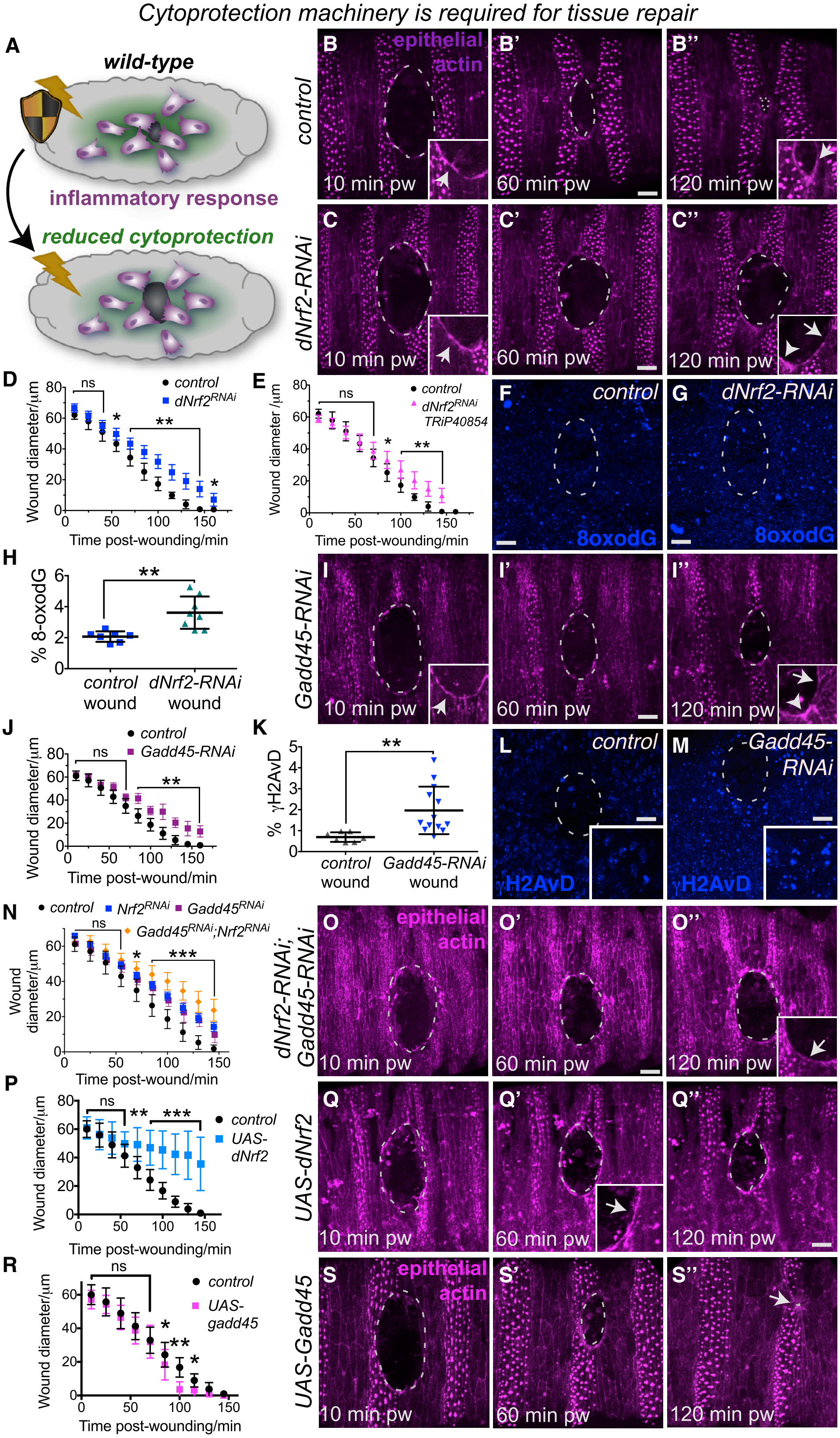

(legend on next page)

down their mRNA targets (Figures S3A and S3B). Simultaneous knockdown of both dNrf2 and Gadd45 caused a further delay in wound repair (Figures 5N and 5O), suggesting that Nrf2 and Gadd45 act synergistically to promote tissue repair. Interestingly, loss of Gadd45γ in Medaka fish also rendered embryos far more sensitive to irradiation-induced DNA damage [33], suggesting that Gadd45's role in stress protection could be conserved in vertebrates.

Given the protective effect conferred by ectopic dNrf2 and Gadd45 to naive tissue (Figure 4), we tested whether overexpression of either dNrf2 or Gadd45 could further accelerate the rate of wound repair. We saw the converse with ectopic expression of dNrf2 throughout the epithelium prior to wounding (using the GAL4-UAS system), which caused marked delays in wound closure (Figures 5P and 5Q). This is consistent with published work that suggests excessive and long-term activation of Nrf2 can have detrimental effects on tissues and may even induce cellular senescence [34, 35]. However, ectopic expression of Gadd45 caused a small but significant increase in the rate of wound closure (Figures 5R and 5S). It is therefore likely that for best therapeutic exploitation of these cytoprotective pathways in the clinic, it would be necessary to transiently activate just prior to surgery to avoid any long-term negative effects (see Discussion).

Wounding Activates a Dynamic Cytoprotective Network of Calcium, JNK, Nrf2, and Gadd45 Signaling

While it is clear that these cytoprotective genes promote tissue resilience and can aid efficient wound repair, what triggers their activation downstream of wounding? The spatial pattern of cytoprotection closely resembles that of the wound-induced calcium wave that spreads out from the injury site within seconds of wounding (Figures 6A and 6B), which we have previously shown to drive inflammatory cell attraction to the wound [17]. We find that JNK signaling is also activated in a similar (but delayed) wave-like pattern at sites of wounding (Figures 6B and 6C; Video S7) using a transgenic reporter of JNK activity (*tre-GFP*) [23], which precedes the wave of Nrf2 reporter activity by approximately 30 min (Figure S4A).

Given their similar activation patterns, we tested whether wound-induced calcium or JNK signaling is important for cytoprotective pathway induction (Figures 6D–6I). Previous work has demonstrated that the *Drosophila* TRPM channel is required for efficient propagation of the wound-induced calcium wave, and RNAi-mediated knockdown of TRPM effectively blocks calcium-mediated inflammatory cell recruitment [17]. Using a similar *trpm-RNAi* approach, we found that inhibition of the wound-induced calcium wave significantly reduced Nrf2 activity (as detected using the ARE-GFP reporter; Figures S4B and S4C) as well as expression of the Nrf2 target GstD1 (Figures 6D and 6E) and Gadd45 (Figures 6F and 6G). Strikingly, this suggests that the induction of epithelial resilience is tightly linked to the pathways that initiate the inflammatory response, suggesting the evolution of a "fail-safe" mechanism for tissue protection at any time or site where inflammation is triggered.

In fact, the upregulation of Gadd45 within the wounded epithelium of both *Drosophila* and murine skin requires input from inflammatory cells, as mutants in both species lacking innate immune cells fail to transcriptionally upregulate Gadd45 at the injury site [27] (Figures S4D and S4E). However, Nrf2 and JNK activation appear to be independent of inflammatory cells as both the GstD1 and JNK reporters were upregulated in a similar spatiotemporal pattern to that of control wounds in *srp* mutants (Figures S4F–S4I). While all cytoprotective pathways are thus activated downstream of the initial (calcium) cue that drives inflammation, some resilience machinery (e.g., Gadd45) also requires input from inflammatory cells themselves.

JNK signaling is activated at wounds in both flies and vertebrates [36, 37], where it stimulates the transcription of genes required for wound closure (such as the actin-binding protein Profilin) [38]. Consistent with this, we find that *Drosophila* wounds completely lacking normal JNK activity (using the dominant-negative JNK, *bsk-DN*) exhibit a marked defect in repair and the wounds remain open for many hours (Figures S4J–S4L). Interestingly, we find that full propagation of wound-induced JNK signaling requires the wound-induced calcium wave as *trpm-RNAi* caused a reduction in the spread of JNK activity (Figure 6H) compared to controls (Figure 6C). Given that wound-induced calcium is known to trigger H_2O_2 production by the NADPH oxidase Duox [17] and that JNK is redox-sensitive [39, 40], we envision that wound-induced JNK signaling could be amplified by epithelial ROS.

JNK signaling has also been linked to the induction of Gadd45 [28], so we tested whether JNK inhibition affected Gadd45 levels post-wounding; JNK inhibition (again using the dominant-negative JNK, *bsk-DN*) also reduced Gadd45 levels in the wounded epithelium (Figure 6I; compared to control, Figure 6F). Full wound induction of Gadd45 thus appears to require signals originating from both within the repairing epithelium (calcium and JNK) and incoming inflammatory cells.

Intriguingly, vertebrate Gadd45β has been implicated in modulating JNK signaling (e.g., in murine hepatocytes [41]),

Figure 5. Wound-Induced Cytoprotective Pathways Are Required for Efficient Wound Repair *In Vivo*

RNAi-mediated inhibition of either *dNrf2* (A, schematic and B–H; independent *dNrf2-RNAi* lines were used in C and D, as in [22], and E, dNrf2-RNAi TRiP40854) or *Gadd45* expression (I–M) caused a delay in epithelial wound repair (C–C″ and quantified in D and E for *dNrf2-RNAi*; I–I″ and quantified in J for *Gadd45-RNAi*, n > 20 for each condition) compared to controls (B–B″; epithelium labeled using Moesin-mCherry in B, C, and I) despite the initial assembly of actin cables at the wound margin (arrowheads, insets, B, C, and I). By 120 min post-wounding, the actin cable had been lost (insets, C″ and I″) compared to controls (inset, B″). Impaired wound healing was associated with elevated levels of oxidative DNA damage (quantified in H; blue, 8-oxo-dG in F and G) following *dNrf2-RNAi* and elevated γH2AvD punctae (quantified in K; blue, γH2AvD in L and M) following *Gadd45-RNAi*. Simultaneous knockdown of dNrf2 and Gadd45 caused more severe delays in wound repair (N and O; inset in O″ indicates loss of actin cable by 120 min post-wounding). Overexpression of dNrf2 significantly delayed wound repair (P and Q) despite assembly of robust actin cable (inset, Q′), but Gadd45 overexpression slightly accelerated wound closure (R and S). pw, post-wounding. % 8-oxo-dG and % γH2AvD refer to percent (%) of area measured that is positive for marker of interest after thresholding. Scale bars represent 10 μm in all panels. Data represented as mean ± SEM; ns, not significant, *p < 0.05 and **p < 0.01 via the Mann-Whitney Test (H and K) or multiple t tests followed by Holm-Sidak multiple comparisons correction (D, E, J, N, P, and R).

See also Figure S3 and Video S6.

CellPress

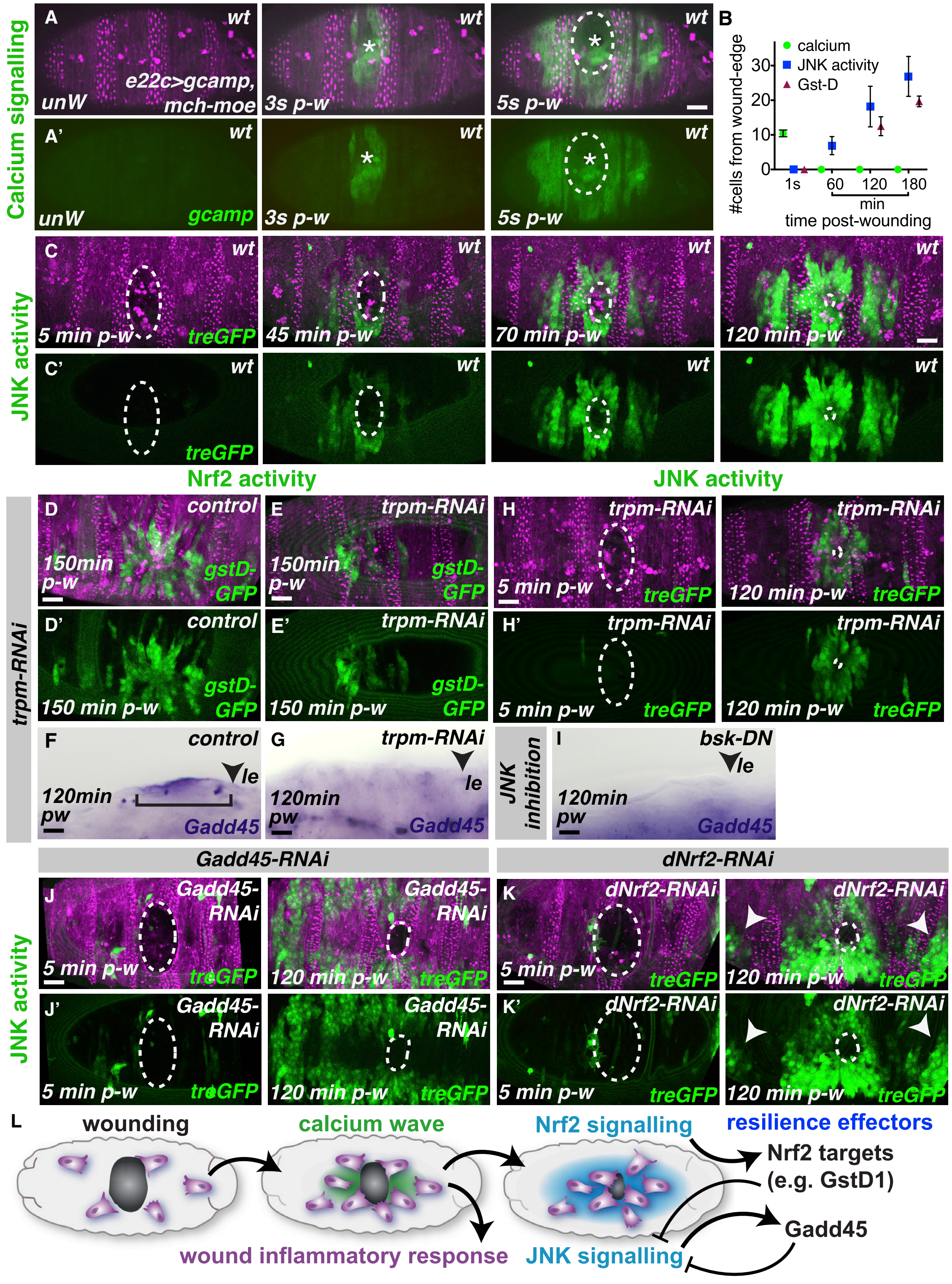

Figure 6. A Complex Network of Wound-Induced Signaling Drives the Expression of Multiple Cytoprotective Factors within the Wounded Epithelium

(A–C) Epithelial (magenta Moesin-mCherry, A) wounding triggers a rapid wave of calcium (green, *Gcamp3* reporter, A and A′) through the epithelium spreading up to 10 cell diameters from the wound edge (B). Wounding also activates JNK signaling (green, *tre-GFP* reporter, C) in the surrounding epithelium (magenta, Moesin-mCherry) but with slower dynamics (B and C).

(legend continued on next page)

leading us to speculate that wound-induced Gadd45 could also feed back on wound-associated JNK signaling. To test this, we analyzed JNK signaling following *Gadd45-RNAi* and found that JNK activity was markedly upregulated (Figure 6J; compared to control, Figure 6C). Given the redox-sensitive nature of JNK signaling [39], we hypothesized that Nrf2 signaling could also restrain JNK activity via its role in ROS detoxification; indeed, loss of *dNrf2* led to ectopic activation of JNK signaling at distances further from the wound site (Figure 6K). Remarkably, this elevated JNK activity was also associated with an increase in Gadd45 expression in areas of the epithelium that normally lack Gadd45 expression (Figures S4O and S4P), consistent with the JNK-dependent induction of Gadd45. To confirm the ROS dependence of JNK activation, we tested whether JNK levels were reduced following the overexpression of ROS scavengers (Catalase; Figure S4M) or when ROS production was inhibited (following Duox-RNAi; Figure S4N); in both cases, JNK activity post-wounding was reduced (Figures S4M and S4N) compared to controls (Figure S4J). Given that sustained and excessive levels of JNK signaling have been linked to apoptosis [42] and could be detrimental to repair, Gadd45 and Nrf2 appear to act together to constrain JNK activity so that it remains at safe pro-regenerative levels within the repairing epithelium (schematic, Figure 6L).

DISCUSSION

Until now, research on cytoprotective factors in wound repair has mainly focused on how antioxidant systems directly minimize ROS-induced damage following injury. However, tissues will undoubtedly have evolved a diverse range of “resilience” mechanisms acting on different cellular targets and working in a highly coordinated manner to collectively reduce damage. In this study, we show that injury activates a cytoprotective signaling network that targets multiple different components to protect the repairing epithelial tissue, including both the upregulation of antioxidant defense machinery and DNA repair mechanisms. In this way, tissue resilience mechanisms can both shield the tissue from damage by directly dampening ROS levels and enhance DNA repair mechanisms (thus making wounded tissues more tolerant to any DNA damage caused by residual ROS). The presence of multiple, partially redundant protective mechanisms ensures effective resilience and thus minimizes delays in tissue repair; indeed, we find that simultaneous knockdown of Nrf2 and Gadd45 exaggerates wound repair defects compared to individual knockouts alone.

Since both Nrf2 and Gadd45α are upregulated within mammalian skin wounds [21, 27], similar networks of wound-induced resilience mechanisms are likely to be well conserved from flies to man. *Drosophila*, with its advanced genetic tractability, capacity for live-imaging, and opportunity for large-scale genetic screening, thus offers an exciting new model for dissecting the mechanisms governing tissue resilience to stress, particularly those during wound repair. Our studies may also have important implications for cancer therapy, as cancer cells could hijack this resilience machinery to protect the tumor from host immune attack, as well as confer resistance to clinical therapies such as chemo- or radiotherapy. Indeed, it is known that Gadd45α deficiency sensitizes epithelial cancer cells to ionizing radiation *in vivo* [43], implicating cytoprotective genes such as Gadd45a as potential drug targets in management of cancer radiotherapy treatments.

For nearly 30 years, experimental biologists and clinicians have observed the remarkable but mysterious phenomenon of “preconditioning,” whereby a brief period of sub-lethal tissue damage triggers adaptive mechanisms that confer subsequent cytoprotection against further insult, either within the same tissue or more remotely [44]. Indeed, recent work in zebrafish has shown that superficial insult (via thoracotomy) preconditions adjacent cardiac tissue and renders it more resilient to subsequent cryoinjury (modeling an infarct) by upregulation of cardioprotective factors [45, 46]. Remarkably, activation of cardioprotective signaling by injection of exogenous ciliary neurotrophic factor just prior to ventricular cryoinjury had beneficial regenerative effects and rendered the heart more resilient to injury [45]. In this regard, therapeutic activation of some or all of these resilience pathways could offer exciting “pre-conditioning” strategies in the clinic to protect patient tissues during surgery or following organ transplant [47].

A better understanding of resilience pathways and their long-term effects (including an analysis of “cost”) is clearly crucial for their full application in a clinical setting, given that excessive and long-term activation of resilience machinery could potentially have adverse effects. Indeed, while we found that ectopic expression of Gadd45 prior to wounding could accelerate wound repair, long-term overexpression of dNrf2 within the epithelium caused marked delays in wound closure. Previous work suggests that prolonged Nrf2 activation may make cells less “competitive” than their neighbors [48] and can also induce certain skin defects (such as hyperkeratosis) [34] and fibroblast senescence [35]. Given the role for wound-induced ROS in inflammatory cell recruitment [3, 17] and angiogenesis [4], we envision that achieving an optimal transient and balanced activation of this endogenous resilience machinery will be the key to unlocking its enormous therapeutic benefits, conferring valuable stress resilience without reaching levels that might otherwise be *detrimental* to repair or later tissue health.

(D–H) Inhibition of the wound-induced calcium wave using *trpm-RNAi* causes reduced expression of the Nrf2 target GstD1 (green, E compared to control in D), loss of Gadd45 expression (purple, *in situ* hybridization; F and G) in the wounded epithelium, and reduced activation of JNK signaling (green, H compared to controls in C).

(I–K) Wound-induced *Gadd45* expression is also lost following inhibition of JNK signaling using *bsk-dominant negative* (arrowhead, I). Wound-induced JNK signaling (green, *treGFP* in J and K) is elevated in areas further from the wound site following RNAi-mediated inhibition of either *Gadd45* (J) or *dNrf2* (K) compared to controls (C).

(L) Schematic illustrates cascading and cross-regulatory network of wound-induced signaling that leads to the upregulation of multiple cytoprotective pathways within the wounded epithelium. Pw, post-wounding; le, leading edge.

Scale bars represent 15 μm in (A), (C), (D)–(F), (J), and (K) and 10 μm in (G)–(I).

See also Figure S4 and Video S7.

STAR★METHODS

Detailed methods are provided in the online version of this paper and include the following:

- KEY RESOURCES TABLE
- LEAD CONTACT AND MATERIALS AVAILABILITY
- EXPERIMENTAL MODEL AND SUBJECT DETAILS
  - *Drosophila* Stocks and Genetics
- METHOD DETAILS
  - Microscopy and Wounding
  - Immunostaining and *In Situ* Hybridization
  - RNA isolation, reverse transcription and real-time qPCR
- QUANTIFICATION AND STATISTICAL ANALYSIS
- DATA AND CODE AVAILABILITY

SUPPLEMENTAL INFORMATION

Supplemental Information can be found online at https://doi.org/10.1016/j.cub.2019.09.035.

ACKNOWLEDGMENTS

We would like to thank members of the Martin, Richardson, and Wood labs for helpful discussion and to Anna Chambers for expert advice. We also thank the Wolfson Bioimaging Facility (University of Bristol, UK), Bloomington Stock Centre (University of Indiana, USA), and Vienna *Drosophila* Resource Centre for *Drosophila* stocks. This work is supported by a Wellcome Trust and Royal Society Sir Henry Dale Fellowship to H.W., an MRC Project Grant to P.M. and W.W. (MR/J002577/1), a Wellcome Trust Senior Research Fellowship to W.W., and a Wellcome Trust Investigator Award to P.M.

AUTHOR CONTRIBUTIONS

H.W. performed the experiments, designed the study, and wrote the manuscript. P.M. and W.W. were initially the principal investigators at the start of the project who, together with H.W., coordinated the project and helped write and copyedit the manuscript.

DECLARATION OF INTERESTS

The authors declare that they have no conflicts of interest.

Received: May 17, 2019
Revised: August 27, 2019
Accepted: September 13, 2019
Published: October 24, 2019

REFERENCES

1. Niethammer, P. (2016). The early wound signals. Curr. Opin. Genet. Dev. *40*, 17–22.
2. Moreira, S., Stramer, B., Evans, I., Wood, W., and Martin, P. (2010). Prioritization of competing damage and developmental signals by migrating macrophages in the Drosophila embryo. Curr. Biol. *20*, 464–470.
3. Niethammer, P., Grabher, C., Look, A.T., and Mitchison, T.J. (2009). A tissue-scale gradient of hydrogen peroxide mediates rapid wound detection in zebrafish. Nature *459*, 996–999.
4. Roy, S., Khanna, S., and Sen, C.K. (2008). Redox regulation of the VEGF signaling path and tissue vascularization: hydrogen peroxide, the common link between physical exercise and cutaneous wound healing. Free Radic. Biol. Med. *44*, 180–192.
5. Martin, P., and Leibovich, S.J. (2005). Inflammatory cells during wound repair: the good, the bad and the ugly. Trends Cell Biol. *15*, 599–607.
6. Warnatsch, A., Tsourouktsoglou, T.-D., Branzk, N., Wang, Q., Reincke, S., Herbst, S., Gutierrez, M., and Papayannopoulos, V. (2017). Reactive oxygen species localization programs inflammation to clear microbes of different size. Immunity *46*, 421–432.
7. Schäfer, M., and Werner, S. (2008). Oxidative stress in normal and impaired wound repair. Pharmacol. Res. *58*, 165–171.
8. Wlaschek, M., and Scharffetter-Kochanek, K. (2005). Oxidative stress in chronic venous leg ulcers. Wound Repair Regen. *13*, 452–461.
9. Dhall, S., Do, D.C., Garcia, M., Kim, J., Mirebrahim, S.H., Lyubovitsky, J., Lonardi, S., Nothnagel, E.A., Schiller, N., and Martins-Green, M. (2014). Generating and reversing chronic wounds in diabetic mice by manipulating wound redox parameters. J. Diabetes Res. *2014*, 562625.
10. Medzhitov, R., Schneider, D.S., and Soares, M.P. (2012). Disease tolerance as a defense strategy. Science *335*, 936–941.
11. Telorack, M., Meyer, M., Ingold, I., Conrad, M., Bloch, W., and Werner, S. (2016). A glutathione-Nrf2-thioredoxin cross-talk ensures keratinocyte survival and efficient wound repair. PLoS Genet. *12*, e1005800.
12. auf dem Keller, U., Kümin, A., Braun, S., and Werner, S. (2006). Reactive oxygen species and their detoxification in healing skin wounds. J. Investig. Dermatol. Symp. Proc. *11*, 106–111.
13. Hausenloy, D.J., and Yellon, D.M. (2016). Ischaemic conditioning and reperfusion injury. Nat. Rev. Cardiol. *13*, 193–209.
14. Razzell, W., Wood, W., and Martin, P. (2011). Swatting flies: modelling wound healing and inflammation in Drosophila. Dis. Model. Mech. *4*, 569–574.
15. Weavers, H., Evans, I.R., Martin, P., and Wood, W. (2016). Corpse engulfment generates a molecular memory that primes the macrophage inflammatory response. Cell *165*, 1658–1671.
16. Wood, W., and Martin, P. (2017). Macrophage functions in tissue patterning and disease: new insights from the fly. Dev. Cell *40*, 221–233.
17. Razzell, W., Evans, I.R., Martin, P., and Wood, W. (2013). Calcium flashes orchestrate the wound inflammatory response through DUOX activation and hydrogen peroxide release. Curr. Biol. *23*, 424–429.
18. Kulms, D., Zeise, E., Pöppelmann, B., and Schwarz, T. (2002). DNA damage, death receptor activation and reactive oxygen species contribute to ultraviolet radiation-induced apoptosis in an essential and independent way. Oncogene *21*, 5844–5851.
19. Ding, A.X., Sun, G., Argaw, Y.G., Wong, J.O., Easwaran, S., and Montell, D.J. (2016). CasExpress reveals widespread and diverse patterns of cell survival of caspase-3 activation during development in vivo. eLife *5*, e10936.
20. Schäfer, M., and Werner, S. (2015). Nrf2–a regulator of keratinocyte redox signaling. Free Radic. Biol. Med. *88* (Pt B), 243–252.
21. Braun, S., Hanselmann, C., Gassmann, M.G., auf dem Keller, U., Born-Berclaz, C., Chan, K., Kan, Y.W., and Werner, S. (2002). Nrf2 transcription factor, a novel target of keratinocyte growth factor action which regulates gene expression and inflammation in the healing skin wound. Mol. Cell. Biol. *22*, 5492–5505.
22. Sykiotis, G.P., and Bohmann, D. (2008). Keap1/Nrf2 signaling regulates oxidative stress tolerance and lifespan in Drosophila. Dev. Cell *14*, 76–85.
23. Chatterjee, N., and Bohmann, D. (2012). A versatile ΦC31 based reporter system for measuring AP-1 and Nrf2 signaling in Drosophila and in tissue culture. PLoS ONE *7*, e34063.
24. Pitoniak, A., and Bohmann, D. (2015). Mechanisms and functions of Nrf2 signaling in Drosophila. Free Radic. Biol. Med. *88* (Pt B), 302–313.
25. Suzuki, T., and Yamamoto, M. (2015). Molecular basis of the Keap1-Nrf2 system. Free Radic. Biol. Med. *88* (Pt B), 93–100.
26. Peretz, G., Bakhrat, A., and Abdu, U. (2007). Expression of the Drosophila melanogaster GADD45 homolog (CG11086) affects egg asymmetric development that is mediated by the c-Jun N-terminal kinase pathway. Genetics *177*, 1691–1702.

27. Stramer, B., Winfield, M., Shaw, T., Millard, T.H., Woolner, S., and Martin, P. (2008). Gene induction following wounding of wild-type versus macrophage-deficient Drosophila embryos. EMBO Rep. *9*, 465–471.
28. Liebermann, D.A., and Hoffman, B. (2008). Gadd45 in stress signaling. J. Mol. Signal. *3*, 15.
29. Plyusnina, E.N., Shaposhnikov, M.V., and Moskalev, A.A. (2011). Increase of Drosophila melanogaster lifespan due to D-GADD45 overexpression in the nervous system. Biogerontology *12*, 211–226.
30. Brand, A.H., and Perrimon, N. (1993). Targeted gene expression as a means of altering cell fates and generating dominant phenotypes. Development *118*, 401–415.
31. Lamers, M.L., Almeida, M.E.S., Vicente-Manzanares, M., Horwitz, A.F., and Santos, M.F. (2011). High glucose-mediated oxidative stress impairs cell migration. PLoS ONE *6*, e22865.
32. Sangani, R., Pandya, C.D., Bhattacharyya, M.H., Periyasamy-Thandavan, S., Chutkan, N., Markand, S., Hill, W.D., Hamrick, M., Isales, C., and Fulzele, S. (2014). Knockdown of SVCT2 impairs in-vitro cell attachment, migration and wound healing in bone marrow stromal cells. Stem Cell Res. (Amst.) *12*, 354–363.
33. Candal, E., Thermes, V., Joly, J.-S., and Bourrat, F. (2004). Medaka as a model system for the characterisation of cell cycle regulators: a functional analysis of Ol-Gadd45γ during early embryogenesis. Mech. Dev. *121*, 945–958.
34. Schäfer, M., Willrodt, A.-H., Kurinna, S., Link, A.S., Farwanah, H., Geusau, A., Gruber, F., Sorg, O., Huebner, A.J., Roop, D.R., et al. (2014). Activation of Nrf2 in keratinocytes causes chloracne (MADISH)-like skin disease in mice. EMBO Mol. Med. *6*, 442–457.
35. Hiebert, P., Wietecha, M.S., Cangkrama, M., Haertel, E., Mavrogonatou, E., Stumpe, M., Steenbock, H., Grossi, S., Beer, H.-D., Angel, P., et al. (2018). Nrf2-mediated fibroblast reprogramming drives cellular senescence by targeting the matrisome. Dev. Cell *46*, 145–161.e10.
36. Rämet, M., Lanot, R., Zachary, D., and Manfruelli, P. (2002). JNK signaling pathway is required for efficient wound healing in Drosophila. Dev. Biol. *241*, 145–156.
37. Okada, Y., Saika, S., Shirai, K., Yamanaka, O., Kitano, A., Wang, Z., Yang, H., and Reinach, P. (2009). JNK MAPK signaling contributes in vivo to injury-induced corneal epithelial migration. Ophthalmic Res. *42*, 185–192.
38. Brock, A.R., Wang, Y., Berger, S., Renkawitz-Pohl, R., Han, V.C., Wu, Y., and Galko, M.J. (2012). Transcriptional regulation of Profilin during wound closure in Drosophila larvae. J. Cell Sci. *125*, 5667–5676.
39. Tobiume, K., Matsuzawa, A., Takahashi, T., Nishitoh, H., Morita, K., Takeda, K., Minowa, O., Miyazono, K., Noda, T., and Ichijo, H. (2001). ASK1 is required for sustained activations of JNK/p38 MAP kinases and apoptosis. EMBO Rep. *2*, 222–228.
40. Finkel, T. (2011). Signal transduction by reactive oxygen species. J. Cell Biol. *194*, 7–15.
41. Papa, S., Zazzeroni, F., Fu, Y.-X., Bubici, C., Alvarez, K., Dean, K., Christiansen, P.A., Anders, R.A., and Franzoso, G. (2008). Gadd45beta promotes hepatocyte survival during liver regeneration in mice by modulating JNK signaling. J. Clin. Invest. *118*, 1911–1923.
42. Liu, J., and Lin, A. (2005). Role of JNK activation in apoptosis: a double-edged sword. Cell Res. *15*, 36–42.
43. Lu, X., Yang, C., Hill, R., Yin, C., Hollander, M.C., Fornace, A.J., Jr., and Van Dyke, T. (2008). Inactivation of gadd45a sensitizes epithelial cancer cells to ionizing radiation in vivo resulting in prolonged survival. Cancer Res. *68*, 3579–3583.
44. Hausenloy, D.J., Barrabes, J.A., Bøtker, H.E., Davidson, S.M., Di Lisa, F., Downey, J., Engstrom, T., Ferdinandy, P., Carbrera-Fuentes, H.A., Heusch, G., et al. (2016). Ischaemic conditioning and targeting reperfusion injury: a 30 year voyage of discovery. Basic Res. Cardiol. *111*, 70.
45. Bise, T., de Preux Charles, A.-S., and Jaźwińska, A. (2019). Ciliary neurotrophic factor stimulates cardioprotection and the proliferative activity in the adult zebrafish heart. NPJ Regen. Med. *4*, 2.
46. de Preux Charles, A.-S., Bise, T., Baier, F., Sallin, P., and Jaźwińska, A. (2016). Preconditioning boosts regenerative programmes in the adult zebrafish heart. Open Biol. *6*, 160101.
47. Lim, S.Y., and Hausenloy, D.J. (2012). Remote ischemic conditioning: from bench to bedside. Front. Physiol. *3*, 27.
48. Kucinski, I., Dinan, M., Kolahgar, G., and Piddini, E. (2017). Chronic activation of JNK JAK/STAT and oxidative stress signalling causes the loser cell status. Nat. Commun. *8*, 136.
49. Kiehart, D.P., Galbraith, C.G., Edwards, K.A., Rickoll, W.L., and Montague, R.A. (2000). Multiple forces contribute to cell sheet morphogenesis for dorsal closure in Drosophila. J. Cell Biol. *149*, 471–490.
50. Brückner, K., Kockel, L., Duchek, P., Luque, C.M., Rørth, P., and Perrimon, N. (2004). The PDGF/VEGF receptor controls blood cell survival in Drosophila. Dev. Cell *7*, 73–84.
51. Ha, E.-M., Oh, C.-T., Ryu, J.-H., Bae, Y.-S., Kang, S.-W., Jang, I.H., Brey, P.T., and Lee, W.-J. (2005). An antioxidant system required for host protection against gut infection in Drosophila. Dev. Cell *8*, 125–132.
52. Greenspan, R. (1997). Fly Pushing: The Theory and Practice of Drosophila Genetics 2nd Revision (Cold Spring Harbor Press).
53. Tautz, D., and Pfeifle, C. (1989). A non-radioactive in situ hybridization method for the localization of specific RNAs in Drosophila embryos reveals translational control of the segmentation gene hunchback. Chromosoma *98*, 81–85.

STAR★METHODS

KEY RESOURCES TABLE

REAGENT or RESOURCE	SOURCE	IDENTIFIER
Antibodies		
anti-γH2AvD	GeneTex	RRID: AB_11165216
anti-8-oxo-dG	Trevigen	RRID: AB_1857195
anti-polyADPribose	BD Biosciences	RRID: AB_394263
anti-GFP	Abcam	RRID: AB_304896
anti-RFP	MBL	RRID: AB_591278
Streptavidin-Cy3	Jackson Immunoresearch	RRID: AB_2337244
Biotinylated anti-mouse	Vector labs	RRID: AB_2687893
Biotinylated anti-rabbit	Vector labs	RRID: AB_2336201
Anti-DIG AP-conjugated antibody	Sigma-Aldrich	RRID: AB_2734716
Bacterial and Virus Strains		
Library Efficiency DH5α Competent Cells	Invitrogen	#18263012
Chemicals, Peptides, and Recombinant Proteins		
Vectashield	Vector labs	RRID: AB_2336789
Heptane	Sigma	#246654
Formaldehyde 37%	Sigma	# 47608
Methanol	Sigma	# 34860
Hydrogen peroxide	Sigma	#95321
Triton-X	Sigma	#X-100
Tween-20	Sigma	# P9416
Trizol	Invitrogen	#15596026
10S Voltalef oil	VWR	#24627.188
DHE	Invitrogen, Molecular Probes	#D11347
H2DCF	Invitrogen, Molecular Probes	#D399
PBS	Sigma	#P5493
Bovine serum albumin	Sigma	#A3983
Proteinase K	Invitrogen	#25530049
Glycine	Sigma	#410225
Denhardts solution	Invitrogen	#750018
NBT	Roche	#11383213001
BCIP	Roche	#11383221001
Durcupan	Sigma	#44610
Critical Commercial Assays		
RNeasy Mini Kit	QIAGEN	# 74104
Thermo Scientific Maxima First Strand cDNA Synthesis Kit	Thermo Scientific	# K1641
PowerUp SYBR Green Supermix	Applied Biosystems	#A25741
Experimental Models: Organisms/Strains		
Drosophila: *ubiquitous-moesin-GFP*	[49]	N/A
Drosophila: *serpent-Gal4*	[50]	N/A
Drosophila: *UAS-GFP*	Bloomington Drosophila Stock Center	RRID: BDSC_6874
Drosophila: *dEcadherin-GFP*	Kyoto Stock Center	#109007
Drosophila: *His2Av-mRFP1*	Bloomington Drosophila Stock Center	RRID: BDSC_23651
Drosophila: *ARE-GFP*	[23]	N/A
Drosophila: *GstD-ARE:GFP*	[22]	N/A
Drosophila: *da-Gal4*	Bloomington Drosophila Stock Center	RRID: BDSC_55851

(*Continued on next page*)

Continued

REAGENT or RESOURCE	SOURCE	IDENTIFIER
Drosophila: *UAS-moesin-mCherry*	[2]	N/A
Drosophila: *OregonR*	Bloomington Drosophila Stock Center	RRID: BDSC_2376
Drosophila: *UAS-Gadd45*	[26]	N/A
Drosophila: *UAS-dNrf2*	[22]	N/A
Drosophila: *UAS-Gadd45-RNAi*	Bloomington Drosophila Stock Center	RRID: BDSC_35023
Drosophila: *UAS-dNrf2-RNAi*	[22]	N/A
Drosophila: *UAS-dNrf2-RNAi*	Bloomington Drosophila Stock Center	RRID: BDSC_40854
Drosophila: *e22c-Gal4*	Bloomington Drosophila Stock Center	RRID: BDSC_1973
Drosophila: *UAS-gcamp3*	[17]	N/A
Drosophila: *TRE-GFP*	[23]	N/A
Drosophila: *UAS-catalase*	Bloomington Drosophila Stock Center	RRID: BDSC_24621
Drosophila: *UAS-Duox-RNAi*	[51]	N/A
Drosophila: *UAS-trpm-RNAi*	Bloomington Drosophila Stock Center	RRID: BDSC_31672
Drosophila: *UAS-basket-DN*	Bloomington Drosophila Stock Center	RRID: BDSC_6409
Drosophila: srp^{3}	[27]	N/A
Drosophila: srp^{AS}	[27]	N/A
Drosophila: *UAS-reaper*	Bloomington Drosophila Stock Center	RRID: BDSC_5824
Oligonucleotides		
dNrf2 F-primer CTGCATCGTCATGTCTTCCAGT	Eurofins Genomics	N/A
dNrf2 R-primer AGCAAGTAGACGGAGCCAT	Eurofins Genomics	N/A
Gadd45 F-primer GGTACTGCTGGAGGCCTTTT	Eurofins Genomics	N/A
Gadd45 R-primer CGCAGTAGTCGACTAGCTGG	Eurofins Genomics	N/A
Rpl32 F-primer AGCATACAGGCCCAAGATCG	Eurofins Genomics	N/A
Rpl32 R-primer TGTTGTCGATACCCTTGGGC	Eurofins Genomics	N/A
Recombinant DNA		
RE38191 cDNA clone	BDGP	# FBcl0207762
Software and Algorithms		
GraphPad Prism V6.01	GraphPad Software	https://www.graphpad.com/scientific-software/prism/
ImageJ/Fiji	National Institutes of Health	https://imagej.nih.gov/ij/
Volocity	PerkinElmer	https://www.perkinelmer.com/lab-products-and-services/resources/cellular-imaging-software-downloads.html
Photoshop	Adobe	https://www.adobe.com/uk/products/photoshop.html
Illustrator	Adobe	https://www.adobe.com/uk/products/illustrator.html

LEAD CONTACT AND MATERIALS AVAILABILITY

Further information and requests for resources and reagents should be directed to and will be fulfilled by the Lead Contact, Helen Weavers (helen.weavers@bristol.ac.uk). This study did not generate new unique reagents.

EXPERIMENTAL MODEL AND SUBJECT DETAILS

Drosophila Stocks and Genetics

Fly stocks were maintained according to standard protocols [52]. All crosses were performed at 25°C unless otherwise stated. The following *Drosophila* stocks were used: *ubiquitous-moesin-GFP* [49], *serpent-Gal4* (*Drosophila* macrophage (hemocyte) specific driver) [50], *UAS-GFP, dEcadherin-GFP, His2Av-mRFP1 (BL23651), ARE-GFP* [23] (4XARE:GFP-16, Nrf2 activity reporter, gift from Ioannis Trougakos), *GstD-ARE:GFP* [22] (ARE of the gstD gene, gift from Ioannis Trougakos), *da-Gal4, UAS-moesin-mCherry* [2], *OregonR, UAS-Gadd45* (gift from Uri Abdu) [26], *UAS-dNrf2* [22] (gift from Ioannis Trougakos), *UAS-Gadd45-RNAi*

(TRiP.HMS01436), *UAS-dNrf2-RNAi* [22] (gift from Ioannis Trougakos), *UAS-dNrf2-RNAi* (TRiP.HMS02021), *e22c-Gal4, UAS-gcamp3* [17], *TRE-GFP* (JNK activity reporter, gift from JP Vincent) [23], *UAS-catalase, UAS-Duox-RNAi* [51], *UAS-trpm-RNAi* (TRiP.JF01465), *UAS-basket-DN, srp*^{3}*, srp*^{AS} and *UAS-reaper*. *Drosophila* mutants and transgenic lines were obtained from the Bloomington Stock Centre unless otherwise stated.

METHOD DETAILS

Microscopy and Wounding

Embryos of the appropriate developmental stage were collected from overnight apple juice plates, dechorionated in bleach for 1 min and mounted on double-sided sticky tape on glass slides in 10S Voltalef oil (VWR). Wounds were induced using a nitrogen-pumped Micropoint ablation laser tuned to 435nm (Andor Technologies) [17]. For ROS detection, dechorionated embryos were microinjected with DHE (Invitrogen, Molecular Probes) or H2DCF (Invitrogen, Molecular Probes) in PBS and then either wounded (as above) or left unwounded for the equivalent time period to be time-matched controls, before mounting and imaging. Microinjections and UV-induced apoptosis were performed as described previously [2, 15]. Prior to microinjection, mounted embryos were dehydrated at room temperature for 5 min prior to covering with oil. Targeted UV exposure was performed using the 405nm laser on the Leica TCS SP5 confocal microscope and the standard integrated FRAP software with continuous bidirectional scanning at 700Hz for 30-180 scans (120 scans used as standard but dose response experiment utilized a range of different scan lengths as detailed in the graphical representation of data). Imaging was performed on a PerkinElmer UltraView spinning disc system or Leica TCS SP5 confocal microscope. Image processing was performed using ImageJ (NIH), Adobe Photoshop or Adobe Illustrator software. For quantification of % area of oxidative and DNA damage, all processing was performed in ImageJ; briefly, confocal images were converted to binary format and thresholded before using the Analyze/Measure tool to calculate the % area.

Immunostaining and *In Situ* Hybridization

Immunostaining was performed using standard techniques with the following antibodies: anti-γH2AvD (rabbit, GeneTex, 1:500), anti-8-oxo-dG (mouse, Trevigen, 1:200), anti-polyADPribose (mouse, LP96-10,1:200), anti-GFP (goat, Abcam, 1:500) and anti-RFP (Rabbit, MBL, 1:500). As for live-imaging experiments, embryos were collected from apple juice plates and dechorionated in bleach. After rinsing with distilled water, dechorionated embryos were fixed for 20 min in a 1:1 heptane and 4% paraformaldehyde (in a phosphate buffer) solution. Following fixation, embryos were devitillinised in 1:1 heptane and methanol by 30 s of vigorous shaking. Embryos were finally rinsed at least three times in methanol and stored at −20°C in fresh methanol until required. Fixed embryos were then blocked in phosphate buffered saline with 0.3% Triton-X detergent and 0.5% bovine serum albumin for one h (PBS-TX-BSA). Embryos were then incubated with diluted primary antibody (at appropriate concentrations) in PBS-TX-BSA overnight at 4°C. The following day, the primary antibody solution was removed and embryos washed three times in PBS-TX-BSA for a total of 30 min before incubation with the appropriate secondary antibody (diluted at 1:200 in PBS-TX-BSA) for one h at room temperature. An extra amplification step was performed where required using biotinylated secondary antibodies and Streptavidin-conjugated fluorophores. Carefully staged embryos were oriented and mounted on a glass slide in Vectashield and imaging was performed on a Leica SP5 confocal microscope. For H_2O_2 treatment, dechorionated embryos were first shaken with a 1:1 mixture of heptane and 100mM H_2O_2 in PBS (or PBS only for controls) for 20min prior to fixation and immunostaining. *Gadd45* RNA localization was performed by *in situ* hybridization using DIG-labeled RNA probes generated by *in vitro* transcription from cDNA templates (RE38191, BDGP). Hybridization and staining was performed according to standard protocols [53]. Fixed embryos were rehydrated in 4% formaldehyde for 30 min, prior to Proteinase K treatment for 2 min (2 μL of a 20mg/mL stock) and 2 brief washes in Glycine (2mg/mL solution) all in PBT buffer (1xPBS and 0.1% Tween20). Embryos were again incubated in 4% formaldehyde for 20 min before transfer to Hybridization buffer containing the appropriate *in situ* probe for overnight incubation at 55°C. The following day, embryos were removed from the hybridization buffer and washed in PBT before a 2 h incubation in anti-DIG AP-conjugated antibody (1/2000 in PBT). After brief rinses in PBT, staining was developed using 4.5 μL NBT and 3.5 μL X-phosphate (BCIP) in 1ml of alkaline phosphatase buffer (100mM NaCl, 50mM $MgCl_2$ and 100mM Tris pH 9.5). Once staining had developed the reaction was stopped by washing in fridge cold PBT. Embryos were then dehydrated using an EtOH series and mounted in Durcupan.

RNA isolation, reverse transcription and real-time qPCR

RNA was isolated from control *da-Gal4*, *da-Gal4* > *UAS-dNrf2-RNAi* and *da-Gal4* > *UAS-Gadd45-RNAi* stage 14/15 embryos by crushing in TRIzol (Life Technologies) and RNA purified using a RNeasy Mini Kit (QIAGEN). Equal quantities of RNA were then reverse transcribed using Thermo Scientific Maxima First Strand cDNA Synthesis Kit and genomic DNA eliminated using ds DNase (Thermo Scientific). Relative quantification of gene expression was performed using PowerUp SYBR Green Supermix with a Real-time PCR machine (QuantStudio Applied Biosystems). *dNrf2* and *Gadd45* gene expression were normalized to the expression of the housekeeping reference gene *rpl32* using the ΔΔCt analysis method. The following primers were used in this study: dNrf2 F-primer CTGCATCGTCATGTCTTCCAGT, R-primer AGCAAGTAGACGGAGCCAT, Gadd45 F-primer GGTACTGCTGGAGGCC TTTT, R-primer CGCAGTAGTCGACTAGCTGG and Rpl32 F-primer AGCATACAGGCCCAAGATCG, R-primer TGTTGTCGATACC CTTGGGC.

QUANTIFICATION AND STATISTICAL ANALYSIS

For quantification of % area of oxidative and DNA damage, all processing was performed in ImageJ; briefly, confocal images were converted to binary format and thresholded before using the Analyze/Measure tool to calculate the % area. All statistical analysis was performed in Prism (Graphpad) as detailed in the legends to each Figure; data represented graphically as mean ± SEM with *$p < 0.05$, **$p < 0.01$ and ***$p < 0.001$ via appropriate statistical tests (such as the Mann-Whitney Test, one-way ANOVA followed by Dunn's multiple comparisons or multiple t tests followed by Holm-Sidak multiple comparisons correction, as described in each Figure Legend).

DATA AND CODE AVAILABILITY

This study did not generate any new computer code or algorithms. The raw confocal imaging datasets supporting the current study are available from the corresponding author on request.

Current Biology, Volume *29*

Supplemental Information

Injury Activates a Dynamic Cytoprotective Network to Confer Stress Resilience and Drive Repair

Helen Weavers, Will Wood, and Paul Martin

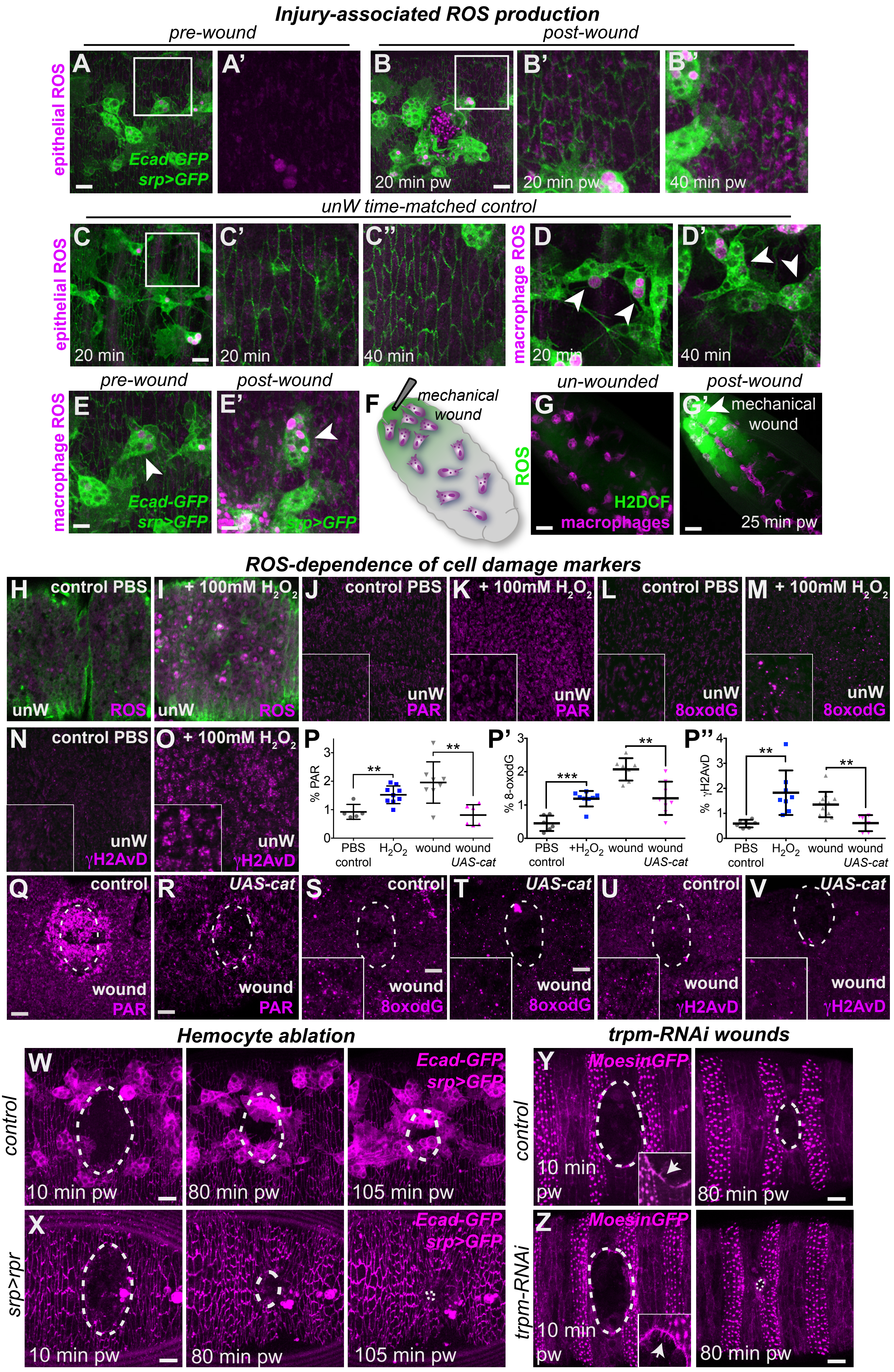
Injury-associated ROS production
pre-wound
post-wound
epithelial ROS
Ecad-GFP
srp>GFP
20 min pw
40 min pw
unW time-matched control
20 min
40 min
macrophage ROS
pre-wound
post-wound
mechanical wound
un-wounded
ROS
H2DCF
macrophages
25 min pw
ROS-dependence of cell damage markers
control PBS
+ 100mM H_2O_2
unW
ROS
PAR
8oxodG
γH2AvD
% PAR
% 8-oxodG
% γH2AvD
PBS control
H_2O_2
wound
wound UAS-cat
control
UAS-cat
wound
Hemocyte ablation
trpm-RNAi wounds
10 min pw
80 min pw
105 min pw
srp>rpr
MoesinGFP
trpm-RNAi

Figure S1. Wounding in *Drosophila* triggers ROS production and the inflammatory response delays healing. Related to Figure 1.

Wounding in *Drosophila* embryos triggers an increase in ROS (magenta, DHE labelling superoxide, A-E) levels within the repairing epithelium (A-B; epithelium labelled with *dE-cadherin-GFP*, green, immune cells ('hemocytes') labelled with *srp>GFP*, green) and within immune cells (E). Images in E and E' are different views of the same embryos in panels A and B'', respectively. Elevated H_2O_2 levels (green, H2DCF) are also observed following wounding of *Drosophila* embryos (F-G', hemocytes labelled with *srp>GFP*, magenta). Unwounded time-matched controls shown for DHE (C-D) and H2DCF (G) staining to control for oxidative conversion of ROS dyes unrelated to wounding. Validation of the ROS-responsive nature of the DNA damage markers (magenta PAR, 8-oxo-dG and γH2AvD) shows that ROS and DNA damage levels are increased following exposure to H_2O_2 (H-O, quantified in P) compared to controls (PBS alone) whilst they are reduced following expression of the Catalase enzyme (Q-V and quantified in P). Inhibition of inflammation, either by genetic ablation of hemocytes (W-X, *srp>reaper*) or *RNAi*-mediated inhibition of *trpm* expression (Y-Z), accelerates wound repair (epithelium labelled using Moesin-mCherry) compared to controls.

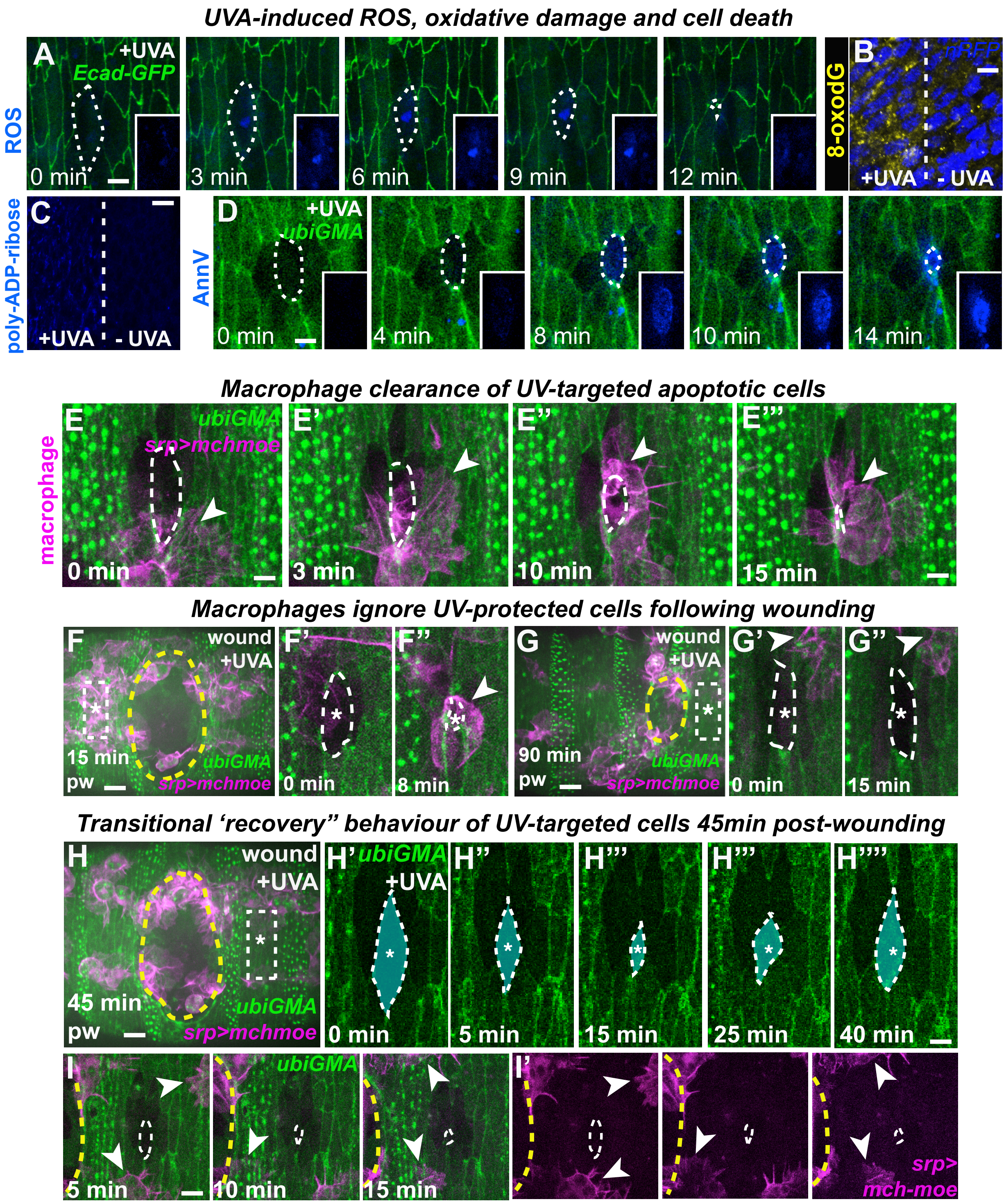
UVA-induced ROS, oxidative damage and cell death
A
+UVA
Ecad-GFP
ROS
0 min
3 min
6 min
9 min
12 min
8-oxodG
B
+UVA
- UVA
C
poly-ADP-ribose
+UVA
- UVA
D
+UVA
ubiGMA
AnnV
0 min
4 min
8 min
10 min
14 min
Macrophage clearance of UV-targeted apoptotic cells
macrophage
E
ubiGMA
srp>mchmoe
0 min
E'
3 min
E''
10 min
E'''
15 min
Macrophages ignore UV-protected cells following wounding
F
wound
+UVA
15 min
pw
ubiGMA
srp>mchmoe
F'
0 min
F''
8 min
G
wound
+UVA
90 min
pw
ubiGMA
srp>mchmoe
G'
0 min
G''
15 min
Transitional 'recovery" behaviour of UV-targeted cells 45min post-wounding
H
wound
+UVA
45 min
pw
ubiGMA
srp>mchmoe
H'
ubiGMA
+UVA
0 min
H''
5 min
H'''
15 min
H''''
25 min
H'''''
40 min
I
5 min
ubiGMA
10 min
15 min
I'
srp>
mch-moe

Figure S2. Wounding in *Drosophila* embryos triggers the induction of stress-resistance. Related to Figure 2.

UV-A treatment of naïve epithelial cells causes a dramatic increase in levels of ROS (blue, DHE, A), 8-oxo-dG (yellow, B; dashed line delineates UVA-exposed region, left from non-exposed, right), poly-ADP-ribose (blue, C) and AnnexinV staining (blue, D). Delaminating epithelial cells (green, ubiquitous GFP-tagged Moesin) targeted with UV-A within naïve unwounded embryos are cleared by migrating hemocytes (magenta, *srp>Moesin-mCherry*, E) as are UV-targeted cells which delaminate within the first 15min post-wounding (F). Epithelial cells within the protected zone that fail to delaminate are ignored by nearby hemocytes (G). Epithelial cells targeted within 45min post-wounding show a transitional behaviour and recover after initial rounding up (H) and are ignored by nearby hemocytes (I). Images in (I) are taken from the same embryo as shown in (H). pw, post-wounding. Scale bars represent 5μm in panels A-E, F'-F'', G'-G'' and H'-H'''' and 10μm in panels F, G and H.

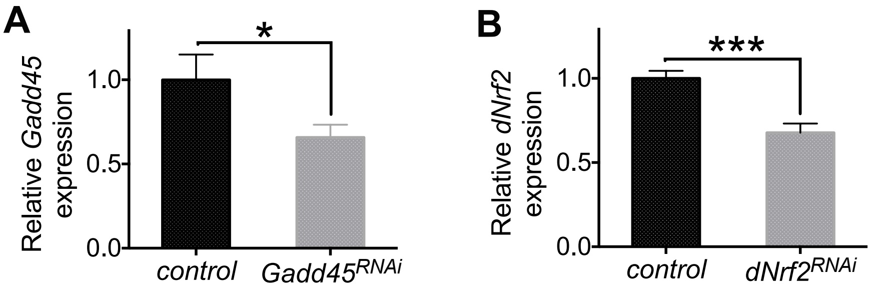
A
B
*
***
Relative Gadd45 expression
Relative dNrf2 expression
1.0
0.5
0.0
control
Gadd45RNAi
dNrf2RNAi

Figure S3. Nrf2 and Gadd45 RNAi significantly reduce expression levels of their respective targets. Related to Figure 5.

RT-qPCR of the relative expression of *dNrf2* (A) and *Gadd45* (B) within whole stage 14/15 embryos following *Gal4* mediated expression of *dNrf2-RNAi* (A) and *Gadd45-RNAi* (B) within the embryonic epithelium; threshold cycle (Ct) values normalised to Rpl32 reveals a significant reduction in *dNrf2* and *Gadd45* expression.

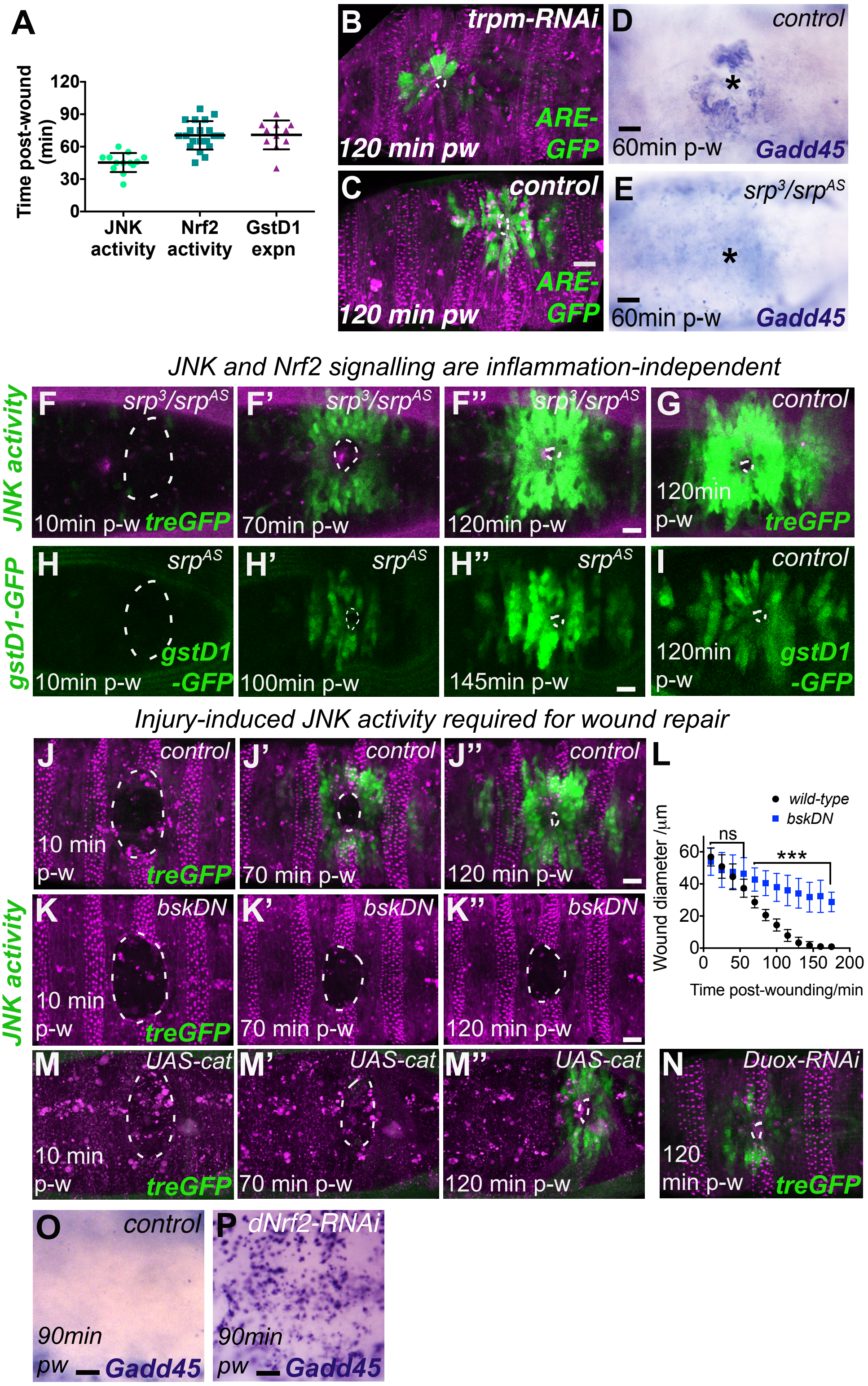

A
Time post-wound (min)
120
90
60
30
0
JNK activity
Nrf2 activity
GstD1 expn
B
trpm-RNAi
120 min pw
ARE-GFP
C
control
120 min pw
ARE-GFP
D
control
60min p-w
Gadd45
E
srp3/srpAS
60min p-w
Gadd45
JNK and Nrf2 signalling are inflammation-independent
JNK activity
F srp3/srpAS
10min p-w treGFP
F' srp3/srpAS
70min p-w
F'' srp3/srpAS
120min p-w
G control
120min p-w
treGFP
gstD1-GFP
H srpAS
10min p-w
gstD1-GFP
H' srpAS
100min p-w
H'' srpAS
145min p-w
I control
120min p-w
gstD1-GFP
Injury-induced JNK activity required for wound repair
J control
10 min p-w
treGFP
J' control
70 min p-w
J'' control
120 min p-w
L
wild-type
bskDN
ns
***
Wound diameter /μm
60
40
20
0
0 50 100 150 200
Time post-wounding/min
JNK activity
K bskDN
10 min p-w
treGFP
K' bskDN
70 min p-w
K'' bskDN
120 min p-w
M UAS-cat
10 min p-w
treGFP
M' UAS-cat
70 min p-w
M'' UAS-cat
120 min p-w
N Duox-RNAi
120 min p-w
treGFP
O control
90min pw
Gadd45
P dNrf2-RNAi
90min pw
Gadd45

Figure S4. A dynamic network of wound-induced signalling pathways drive tissue resilience. Related to Figure 6.

JNK signalling is activated in response to wounding (A, time at which reporter activity first detected within repairing epithelium), prior to that observed for Nrf2 signalling and GstD1 expression. *Trpm-RNAi* mediated inhibition of the inflammatory calcium wave reduced activation of the Nrf2 activity reporter *ARE-GFP* (green, B-C) around the wound site (epithelium magenta, Moesin-mCherry). Wound-induced expression of *Gadd45* (control, D) is reduced in *srp* mutant embryos that lack a wound-induced inflammatory response (E). Inflammation *per se* is not required for wound-induced activation of JNK (green, *tre-GFP*, F-G) or GstD1 expression (green, *gstD-GFP* reporter, H-I) as JNK and GstD1 reporter activity resembles that of control embryos (G and I, respectively) in *srp* mutants. JNK signalling (green, *tre-GFP*, J), activated in response to wounding, is required for efficient wound repair in *Drosophila* embryos as inhibition of JNK signalling using *UAS-basket-dominant-negative* (K) causes a significant delay in wound closure (K and quantified in L). JNK signalling is responsive to ROS levels as expression of Catalase (M) or Duox-RNAi (N) reduced the activation of the *treGFP* JNK reporter. RNAi-mediated inhibition of dNrf2 expression caused elevated Gadd45 expression (P) in regions of the epithelium that normally lack Gadd45 (O). pw, post-wounding. Data in represented as mean ± SEM; *$p < 0.05$, **$p < 0.01$, ***$p < 0.001$ via the multiple t-tests followed by Holm-Sidak multiple comparisons test (L). Scale bars represent 10μm in panels B-P.
